# Advancing Plastic Recycling: Challenges and Opportunities in the Integration of 3D Printing and Distributed Recycling for a Circular Economy

**DOI:** 10.3390/polym15193881

**Published:** 2023-09-25

**Authors:** Ali Kassab, Dawood Al Nabhani, Pravansu Mohanty, Christopher Pannier, Georges Y. Ayoub

**Affiliations:** 1 Department of Industrial and Manufacturing Systems, University of Michigan-Dearborn, Dearborn, MI 48128, USA; akassab@umich.edu; 2 Department of Mechanical Engineering, University of Michigan-Dearborn, Dearborn, MI 48128, USA; dalnabha@umich.edu (D.A.N.); pannier@umich.edu (C.P.)

**Keywords:** thermoplastics, mechanical recycling, circular economy, distributed recycling, additive manufacturing

## Abstract

The concept of the circular economy has emerged as a promising solution to address the mounting concerns surrounding plastic waste and the urgent need for sustainable resource management. While conventional centralized recycling remains a common practice for plastic waste, centralized facilities may prove inadequate in handling the ever-increasing volumes of plastic waste generated globally. Consequently, exploring alternative recycling methods, such as distributed recycling by additive manufacturing, becomes paramount. This innovative approach encompasses actively involving communities in recycling practices and promotes a circular economy. This comprehensive review paper aims to explore the critical aspects necessary to realize the potential of distributed recycling by additive manufacturing. In this paper, our focus lies on proposing schemes that leverage existing literature to harness the potential of distributed recycling by additive manufacturing as an effective approach to plastic waste management. We explore the intricacies of the recycling process, optimize 3D printing parameters, address potential challenges, and evaluate the mechanical properties of recycled materials. Our investigation draws heavily from the literature of the last five years, as we conduct a thorough critical assessment of DRAM implementation and its influence on the properties of 3D printing structures. Through comprehensive analysis, we reveal the potential of recycled materials in delivering functional components, with insights into their performance, strengths, and weaknesses. This review serves as a comprehensive guide for those interested in embracing distributed recycling by additive manufacturing as a transformative approach to plastic recycling. By fostering community engagement, optimizing 3D printing processes, and incorporating suitable additives, it is possible to collectively contribute to a more sustainable future while combatting the plastic waste crisis. As progress is made, it becomes essential to further delve into the complexities of material behavior, recycling techniques, and the long-term durability of recycled 3D printed components. By addressing these challenges head-on, it is feasible to refine and advance distributed recycling by additive manufacturing as a viable pathway to minimize plastic waste, fostering a circular economy and cultivating a cleaner planet for generations to come.

## 1. Introduction

The utilization of plastic materials in various structural and non-structural applications has witnessed significant growth over the past 70 years, attracting interest across multiple industries. However, a major concern arises from the fact that most plastic produced in the last 60 years is not biodegradable and, consequently, can take decades to decompose [1,2,3]. With a staggering 86% of plastic ending up in landfills [4], a substantial volume of plastic waste has accumulated over the last few decades, resulting in waste-management issues as well as the environmental crises of microplastics in marine environments and microplastic ingestion by humans and animals [5,6]. One key issue contributing to this problem is the prevalence of plastics intended to be discarded after a single use. Instead of single-use, plastics could be given new life through recycling to make use of the embodied energy already spent in production and distribution of the plastic [7,8]. Therefore, it becomes crucial for designers to consider in their material selection and product design, the recycling of the material for use in applications different from the initial application. As a note to the reader, it is important to clarify that in this manuscript, the terms “thermoplastics” and “plastics” will be used interchangeably to refer only to the polymers that can be reprocessed by melting.

Plastic waste poses significant challenges for effective recycling. Plastic materials constitute a substantial portion, approximately 12.9%, of total US municipal solid waste [9]. Unlike metals, which are relatively easier to recycle, plastic waste presents unique difficulties. The production of plastic materials encompasses a wide range of applications, with packaging and single-use products accounting for approximately 50% of its usage. In contrast, only a modest proportion, between 20% and 25%, of produced plastics are employed in long-term infrastructure, primarily in the construction and building sectors. The remaining plastic production caters to intermediate lifespan products including electrical and electronic goods, furniture, vehicles, and agriculture [2,10,11]. Plastic production is distributed across various types of polymers: polyethylene (PE)-based products constitute 24% of plastic production, followed by polypropylene (PP) products at 16.6%, polyvinyl chloride (PVC) products at 11.4%, polyurethane (PU) products at 5.5%, polystyrene (PS) products at 6.1%, and polyethylene terephthalate (PET) products at 5.3% [12]. The combination of different polymers and the potential contamination by food particles, metal, paper, pigment, and ink further complicates the recycling of polymers. The challenges in plastic recycling are exacerbated by a shortage of collection and sorting facilities, the difficulty in effectively sorting different types of plastic, and the high cost associated with collecting and processing plastic waste. The higher costs associated with plastic recycling act as a deterrent for manufacturers and investors alike. This issue presents a significant barrier that hinders the widespread adoption of plastic recycling initiatives. Recycling and remanufacturing plastic can save between 30% to 80% of carbon emissions compared to processing and manufacturing of virgin plastics [13]. As governments implement carbon pricing, the economics of plastic recycling will improve compared to virgin plastic production. To enhance the quality of recycled polymers and increase their economic viability as alternatives to virgin materials, significant investment in recycling technologies is necessary.

The degradation rates of polymers are significantly influenced by environmental factors such as ultraviolet light, oxygen, and temperature. These environmental influences pose challenges to recycling efforts and contribute to the overall decrease in the quality of recycled plastic material. Compared to their non-recycled counterparts, recycled plastics often exhibit lower quality, characterized by color variation and decreased strength. These quality issues pose barriers to their utilization in the manufacturing sector, as they may be less appealing to manufacturers and hinder their incorporation into new products [2]. The compromised quality of recycled plastics necessitates further attention and research to address these limitations and enhance the desirability and applicability of recycled materials in various industries.

Plastic recycling encompasses various methods, classified into four categories: primary recycling, secondary recycling, tertiary recycling, and quaternary recycling. Each category involves distinct processes and objectives. Primary recycling, also known as closed-loop recycling, entails mechanically reprocessing plastic scrap to produce a product with properties equal to those of the original material. Secondary recycling, referred to as downgrading recycling, involves mechanically reprocessing plastic scrap to produce a product with altered properties. Tertiary recycling focuses on the recovery of chemical constituents from plastic scrap, while quaternary recycling harnesses the energy content of scrap plastic to generate steam and electricity [2]. Mechanical recycling is used in both primary and secondary recycling. While most thermoplastics, including PET, PE, and PP, have high potential for mechanical recycling, thermosets such as unsaturated polyester and epoxy resin cannot be mechanically recycled due to their molecular structure. The varying processing requirements and molecular incompatibility of different plastic types present challenges in the production of recycled plastic from plastic waste. The mechanical recycling process comprises several key stages, including collection, sorting, cleaning, size reduction, and compatibilization or separation. Typically, these stages occur at centralized recycling facilities strategically located near industrial areas or urban centers. These large-scale recycling centers are equipped with specialized machinery and technologies capable of handling significant volumes of plastic waste, enabling efficient processing.

**Figure 1:**
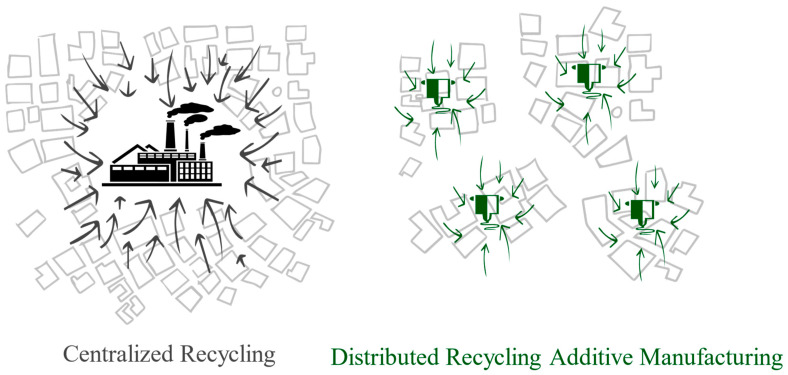
Comparing Centralized Recycling to Distributed Recycling by Additive Manufacturing.

However, despite the existing infrastructure and centralized recycling facilities, the traditional recycling model faces certain challenges that limit its effectiveness. These challenges include the scarcity of collection and sorting facilities, difficulties in separating different plastic types, and the high costs associated with collecting and processing plastic waste. Furthermore, the low weight-to-volume ratio of plastic waste exacerbates the economic feasibility of the traditional recycling process. As an illustration, Craighill and Powell [14] highlight that PET plastics generated in Britain are often exported to centralized recycling facilities in the Netherlands and Ireland for recycling, reflecting the challenges posed by transporting lightweight plastic waste over long distances. The reliance on centralized facilities and the associated logistics of transporting waste materials from individual consumers to recycling centers and back to manufacturers are increasingly considered impractical and environmentally burdensome. Despite comprising less than 5 percent of the worldwide population in 2016, North America was responsible for producing 14 percent of the planet’s total waste, amounting to 289 million tons, translating to a daily rate of 2.21 kg per person. The high collection rate of waste in North America, at 99.7 percent, is attributed to sufficient funding for waste management. Nevertheless, 12 percent of this waste is incinerated, over half ends up in landfills, and only about one-third is subjected to recycling. Part of this challenge is not solely driven by costs but rather by the complexities within the logistics network [15]. To establish an efficient and sustainable plastic recycling ecosystem, it is imperative to adopt a comprehensive approach that integrates localized recycling solutions alongside centralized facilities. This approach aims to reduce the reliance on new resources and minimize waste production, while simultaneously improving energy efficiency and promoting environmental sustainability. These objectives align with the principles of a circular economy, which seeks to protect the environment, foster social justice, and encourage sustainable economic growth. By embracing a comprehensive approach, the plastic recycling ecosystem can transition from a linear model to a circular one, in which materials are kept in use for as long as possible and waste is minimized. Further, this transformation requires localized recycling solutions that are close to the point of waste generation to reduce the need for extensive transportation and logistics. The integration of localized recycling into the existing network of centralized facilities offers the potential to create a more resilient and adaptable system that is responsive to the unique needs of different communities and regions. A promising localized approach that promotes resource efficiency, minimizes environmental impacts, and fosters a more sustainable and inclusive future for plastic recycling is the concept of distributed recycling, illustrated in Figure 1. Figure 1 presents two distinct recycling models. The first model represents the existing centralized recycling facilities, wherein plastic waste is collected from across a large area and transported to a central facility for processing. While this model has been effective to date, it faces limitations from transportation costs and the commensurate energy expended and carbon emissions. In contrast, the second model shows distributed recycling. Operating on a smaller scale, this system runs in parallel with the established centralized system, contributing to a more decentralized and locally driven recycling process. In this distributed model, recycling takes place closer to the source of waste generation, reducing the need for extensive transportation and allowing for greater community involvement.

Defining the circular economy in precise terms can be challenging due to the many facets of the concept [16,17]. However, at its core, the circular economy seeks to provide an alternative to the linear economic model, which is inherently unsustainable. The circular economy prioritizes the circularity of resource flow and aims to prevent the loss of materials from the system [18]. While recycling is often associated with circularity efforts, it is just one aspect of the broader circular economy framework. The circular economy takes a systemic approach that integrates economic, environmental, and social sustainability principles. It aims to maintain the highest value of products and materials within the system for as long as possible, reduce reliance on non-renewable resources, minimize waste generation from the outset, and prevent contamination, toxicity, and pollution. Unlike traditional recycling, which primarily focuses on mitigating environmental damage and pollution, the circular economy aims to address the underlying causes of environmental degradation [19]. To achieve a more sustainable and effective approach to plastic recycling, it is crucial to prioritize local or regional solutions over the global waste trade. This entails implementing circularity strategies that limit the geographic reach of end-of-life plastic products. By doing so, the negative environmental impacts associated with long-distance transportation of plastic waste can be minimized. Additionally, this approach fosters the development of robust and transparent local recycling systems that are better equipped to meet the unique needs of individual communities [20]. Embracing the principles of the circular economy in the context of plastic recycling can lead to substantial benefits. It decreases the dependence on virgin resources, reduces waste generation, promotes resource efficiency, and contributes to the creation of a more sustainable and resilient recycling ecosystem. By prioritizing local solutions and integrating circularity strategies, it is achievable to strive towards a future in which plastic waste is minimized, and materials are continually circulated within the economy, aligning with the principles of environmental preservation and social well-being.

A promising development in polymer recycling involves the integration of additive manufacturing facilities with relatively smaller-sized plastic extruders [21]. Additive manufacturing, commonly known as 3D printing, is a transformative technology that constructs structures layer by layer to create three-dimensional objects. This process has gained significant attention for its ability to create complex structures with high precision and customization. In the context of plastic recycling, additive manufacturing can be integrated with recycling processes, giving rise to a novel approach known as distributed recycling by additive manufacturing (DRAM). DRAM represents a paradigm shift in traditional recycling practices by combining the benefits of additive manufacturing with recycling principles, offering the potential to make recycling more accessible and participatory. DRAM enables decentralized recycling schemes that operate closer to the point of use, offering numerous advantages over centralized recycling systems. One key advantage of DRAM is the elimination of the need to manage and operate a centralized system with physical inventories, along with the associated transportation costs of shipping waste to a central recycling facility. By enabling recycling to occur locally, DRAM simplifies supply chain systems and logistics [21,22]. Moreover, the integration of additive manufacturing in DRAM opens up possibilities for producing complex structures, enabling innovative approaches to open-loop recycling [23]. This integration facilitates the engagement of a broader community in the recycling process, resulting in a larger volume of waste being reutilized. While the distributed recycling initiative remains a dynamic project with its exact metrics yet to be fully determined, it surely complements and addresses the shortcomings of a centralized system aimed at achieving economies of scale and harnessing advanced technology. In conjunction with the existing approach, this scheme fosters community involvement, minimizes transportation needs, and fortifies the resilience of the recycling system against potential disruptions.

The practice of open-loop recycling plays a crucial role in maintaining resource flow and advancing the establishment of a circular economy [7]. Utilizing recycled plastic waste as 3D printing materials is an option that is financially viable and easily achievable. As a consequence, the cost of 3D printing materials will decrease, and the wide adoption of this recycling practice has a ripple effect on reducing the prices of 3D printers themselves. Recent years have seen a significant decline in the prices of 3D printers, providing clear evidence of the cost reduction associated with their widespread use. In 2014, a typical consumer-grade 3D printer cost approximately $1000. However, just two years later, in 2016, due to a remarkable compound annual growth rate of 30%, equivalent to a billion-dollar increase each year, the average price of budget-friendly 3D printers plummeted to less than $400 [24,25]. At present, affordable 3D printers designed for home use, while possibly lacking certain advanced features such as enhanced printing precision sensors and automation, are ideally suited for recycling purposes and are available within the price range of $100 to $400. It is reasonable to assume that an increased demand for 3D printers, driven in part by recycling efforts, could further contribute to reducing their prices. This substantial decrease in price effectively eliminates barriers for using 3D printing technology, making it accessible to a broader audience, including individuals, businesses, schools, community centers, and households. These communities are becoming essential participants in the effort to incorporate recycled waste into their printing materials [26]. Moreover, sectors such as the military can benefit from 3D printing by reducing their dependence on complex supply chains and avoiding operational delays. The US military, for instance, has shown interest in using recycled plastic bottles collected in camps to create replacement supplies for soldiers on the battlefield, providing a sustainable and conservation-focused alternative [27].

The integration of additive manufacturing and distributed recycling not only offers environmental benefits but also fosters local empowerment and engagement in the recycling system. By decentralizing the recycling process and involving a broader range of stakeholders, it is possible to unlock the potential for increased waste reutilization, resource conservation, and the establishment of a more circular economy.

However, achieving high-quality structures with 3D printing requires tuning process settings [28]. Factors such as wall thickness, infill density, and temperature control play crucial roles in determining the strength and durability of the printed parts. Moreover, the polymeric waste used for recycling often undergoes various forms of degradation, including contamination, exposure to environmental elements, and the application of shear forces and high temperatures, which can significantly affect the quality of the recycled materials. Therefore, it is imperative to conduct further research to comprehensively understand the mechanical properties of recycled thermoplastic polymers when employed in additive manufacturing [29]. This knowledge can inform the development of guidelines and best practices for 3D printing with recycled materials, ensuring that the resulting structures meet necessary quality standards. By educating local communities on the use of 3D printing in recycling and fostering their active involvement in the process, it is possible to create a more inclusive and sustainable recycling ecosystem. Empowering communities with knowledge and skills related to additive manufacturing and recycled materials will not only drive local economic development but also contribute to global efforts in promoting environmental sustainability. Through collaborative efforts and informed practices, there is potential to harness the potential of 3D printing in recycling to advance towards a more resource-efficient and environmentally conscious future.

To gain a broader understanding of the existing body of research on plastic recycling, Table 1 offers a comprehensive summary of published review articles that have extensively explored this topic. Additionally, it highlights the key findings and conclusions derived from these previous reviews. Previous research has explored the integration of 3D printing in plastic recycling, considering various approaches and examining economic and environmental aspects. However, these studies have only provided a cursory overview of the challenges and opportunities associated with this method. Conversely, other research has focused on the broader challenges of recycling without specifically addressing 3D printing. To fully understand and integrate 3D printing as a complementary approach within the circular economy of plastics, a comprehensive investigation of the distinct challenges and opportunities of 3D printing recycling is necessary. Given the limited available sources on this topic, this review paper aims to bridge this knowledge gap by discussing the current challenges and insights from other mechanical recycling methods. It then delves into a more detailed examination of 3D printing recycling. Furthermore, the paper intends to explore the mechanical properties of recycled polymers by drawing upon existing literature on 3D printing and injection molding. This comprehensive approach will enhance our understanding of these properties and enable the identification of necessary conditions for successful 3D printing recycling experiments. To achieve these goals, the paper is structured as follows: Section 2 addresses the challenges encountered throughout the multiple stages of recycling commonly conducted in centralized facilities. This discussion provides an understanding of the existing obstacles in traditional recycling practices. Section 3 introduces the concept of 3D printing and distributed recycling within the context of the circular economy, establishing a connection between the challenges outlined in the previous section and the potential issues that may arise in this alternative approach. By exploring the implications of adopting 3D printing for distributed recycling, it is possible to identify how it addresses or introduces new challenges in the recycling process. Section 4 examines potential strategies to enhance the mechanical properties of recycled polymers. While previous research has predominantly focused on such improvements in relation to injection molding, this exploration opens new avenues for investigating chemicals and agents specifically applicable to 3D printing recycling. Finally, the paper concludes with a summary that outlines the research areas that should be prioritized to effectively implement 3D printing recycling in the circular economy of plastics, emphasizing the need for further investigation and development. By following this structured approach, this review paper aims to provide a comprehensive understanding of the challenges, opportunities, and potential improvements in 3D printing recycling. It serves as a foundation for future research and development, guiding efforts to advance the integration of 3D printing in the circular economy of plastics.

## 2. Plastic Mechanical Recycling: Processes and Challenges

Mechanical recycling is a multi-step process that involves several key stages, including collection, sorting, cleaning, shredding, and compatibilization or separation. However, the ease of recycling varies depending on the types of plastic, with some posing greater challenges than others. While closed-loop recycling is theoretically possible for most thermoplastics, practical implementation presents financial and technical difficulties. The complexities arising from different processing requirements and molecular incompatibilities between plastic types make the production of recycled resins from plastic waste a complex task. Despite these challenges, mechanical recycling plays a vital role in waste management by conserving natural resources, reducing greenhouse gas emissions, and diverting waste from landfills. To enhance the effectiveness of mechanical recycling, ongoing research and development efforts focus on developing new technologies, improving sorting methods, and enhancing compatibility among different plastic types. Furthermore, consumer education and the implementation of effective recycling programs and policies are crucial to advancing mechanical recycling efforts.

Mechanical recycling encompasses both primary and secondary recycling approaches. Closed-loop recycling, also known as primary recycling, involves the repurposing of post-consumer plastic materials, such as industrial parts or single-use plastic, to create new products with similar properties. This recycling process aims to achieve a closed loop, where the recovered material is reintroduced into its original application, maintaining a circular flow of resources (e.g., recycling post-consumer PET bottles into new bottles). However, closed-loop recycling isn’t extensively embraced by recyclers due to the need for partially clean scrap plastics. On the other hand, secondary recycling focuses on the mechanical reprocessing of more complex or contaminated plastics than those encountered in closed-loop recycling. The resulting recovered plastic is typically used in products with lower performance requirements compared to the original application. These products do not need to meet the same stringent standards or undergo the same level of usage as those made from virgin materials.

**Figure 2:**
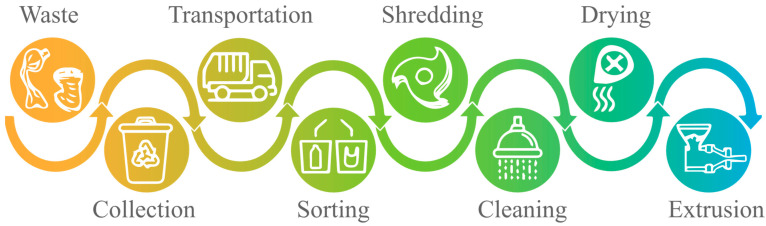
Stages of Plastic Mechanical Recycling Process.

Mechanical recycling presents significant challenges at various stages, demanding effective collection, efficient sorting, and a thorough understanding of properties and behavior of diverse plastic types. The presence of contaminants or different types of plastics, especially in multi-layer plastics, further complicate sorting and reprocessing. Hence, there is an urgent need to tackle these challenges by increasing recycling efficiency and recycled end-product quality. Figure 2 illustrates the stages involved in standard recycling procedures. Table 2 provides a comprehensive overview of recent studies (within the past 5 years) that investigated the various stages of plastic recycling, with a specific emphasis on the challenges encountered at each stage.

### 2.1. Waste Collection and Sorting for Recycling

The initial stage of the mechanical recycling process involves the collection of post-consumer materials from households, commercial establishments, and institutions. Effective management of plastic waste requires tailored waste collection methods based on different types of waste and population densities [88]. In densely populated areas where a significant volume of waste is generated, daily household waste collection using specialized vehicles has proven to be an effective method [89]. These vehicles are equipped with specialized equipment to collect, compact, and transport waste to designated waste management facilities. This approach is particularly suitable for densely populated areas where waste accumulates rapidly. Conversely, in areas with lower population densities, cost-effective solutions involve placing bins in the locality that are emptied regularly. This method is suitable for areas where the waste volume does not justify daily waste collection using vehicles. Drop-off recycling is another effective method for managing plastic waste [90]. It involves providing large bins or machines in easily accessible areas such as community centers or nearby residential areas. Residents can conveniently drop off their plastic waste into the designated bins. This method works well for recycling specific types of plastic waste, such as bottles or packaging. Buy-back centers offer fixed or mobile options for exchanging waste for useful items. These centers incentivize residents to recycle their plastic waste by providing rewards in the form of useful items, household goods, or vouchers for local stores [91]. This approach encourages community participation in recycling efforts. Another noteworthy collection method is the deposit or refund system, where waste packaging can be returned for recycling in exchange for credits, cash, or tax returns. This method provides residents with financial incentives to recycle their plastic waste and is particularly effective in countries with low recycling rates, as it offers tangible benefits that encourage participation. By employing diverse collection methods tailored to specific contexts, communities can enhance waste management and recycling practices, promoting a more sustainable approach to plastic waste.

The last two methods of plastic waste collection mentioned above rely on providing incentives to individuals, recognizing the significant obstacle of raising community awareness and motivation. Insufficient knowledge on proper plastic waste sorting further compounds this challenge, stemming from a lack of clear guidance and readily available information on recyclable plastics. Moreover, the process of sorting plastic waste can be time-consuming and inconvenient, discouraging individuals from engaging in proper waste sorting practices. Additionally, the lack of infrastructure for managing plastic waste presents a significant obstacle in numerous countries worldwide, particularly those in developing regions. In these areas, the collection and disposal of plastic waste is further complicated by weak policies and a shortage of resources [92].

Following the waste collection process, the next crucial step in the recycling process is the “End-of-Waste” phase [35], where waste materials are transformed into new raw materials before being reprocessed into new products. The initial stage of this phase involves the separation and categorization of recyclable materials, which is particularly important due to the immiscibility of thermoplastic polymers, causing different plastic types to be incompatible with one another [2]. Sorting plastics is a critical step in the recycling process and requires various complementary methods. One such method is manual sorting, where skilled operators sort materials according to their type. Despite its high effectiveness, the manual approach can be expensive. Several density separation methods are employed in sorting, including float–sink separation. This technique involves using water to float shredded flakes of polymers with densities below 1 g/cm^3^, allowing for their separation. Froth flotation is a particle separation technique based on differences in surface properties. The process includes introducing bubbles into a mixture of particles and water, where specific particles adhere to the bubbles and ascend to the surface, resulting in the formation of a froth layer. Optical sorting of plastics relies on the use of lasers, cameras, and sensors to identify and sort plastics based on their physical properties, such as color, transparency, and reflectivity. X-ray technology is also utilized to sort plastics based on their elemental composition, which is particularly useful for identifying and separating PVC containers due to their high chlorine content [35,93,94]. However, this method may not be suitable for sorting large quantities of plastic, as it can lead to potential misclassification. Spectroscopy is commonly employed for sorting bottles in commercial industries, but has limitations when used for sorting other plastic products. This is due to the incompatibility of the radiation technologies used to identify the plastic’s chemical structure. For instance, durable goods such as automobiles and appliances exhibit significant variations in shape, size, and thickness, making it challenging to find a suitable orientation that accurately identifies the plastic and transmits the energy through its thick walls [95].

The presence of certain additives, pigments, and reinforcements in plastic products further complicates the sorting process. Carbon black pigments, for example, can block spectroscopic scanning and reduce the efficiency of sorting [96]. Black plastics have been identified as a hindrance to efficient sorting [97,98]. Additionally, reclaimers have reported that other colors of plastics can result in up to 35% of plastics being classified incorrectly. Furthermore, the variety of additives in durable products, such as automobiles and appliances, increases the complexity of sorting and reduces the accuracy of identification systems. Moreover, sorting contaminated waste presents an added layer of difficulty, not only due to its detrimental impact on the material’s properties but, more importantly, because of the health hazards it poses.

Addressing chemical contamination is crucial for achieving high-quality recycled plastics. Strategies such as improved sorting and separation techniques, proper cleaning methods, and careful consideration of plastic compatibility during recycling can significantly mitigate the adverse effects of chemical contamination. By focusing on these aspects, it is feasible to foster the development of a more sustainable and effective plastic recycling process.

Chemical contamination poses a significant threat to the microstructural and chemical properties of post-consumer plastics. The quality of plastics can vary depending on their specific applications and intended uses [99]. Plastics used in food packaging, for example, are subject to stringent regulations regarding their chemical composition and the potential release of harmful substances. Conversely, plastics not utilized in the food industry may contain a range of chemical additives in varying concentrations. Moreover, unintended substances, such as residues from catalysts or metal impurities from non-metal additives, can be introduced during the production process. The challenge is further exacerbated as some contaminants become chemically embedded in the plastic matrix, making simple washing insufficient to remove them, resulting in their retention even after recycling [100]. Additionally, most plastic types are incompatible with one another at a molecular level and have diverse processing requirements. Consequently, contamination may arise when different plastics chemically react with elements present in the other’s matrix. According to Hopewell et al. [2], even a small quantity of PVC contaminant in a PET recycling process can result in the degradation of the recycled PET. This degradation occurs due to the release of hydrochloric acid gas from the PVC at the higher temperature needed to melt and reprocess PET. Consequently, the recycled PET may exhibit issues such as brittleness, yellowing, and diminished adhesive properties.

Ink and coatings (such as paint) are significant chemical substances that can be found on plastics. Ink, when present, can reduce the transparency and may result in chemical reactions that weaken the plastic’s mechanical properties. On the other hand, coatings on plastics create a twofold problem during the recycling process. Firstly, certain coatings can act as stress risers in specific applications. Secondly, the degradation of the coating itself adds to the deterioration of the plastic material [98].

Contamination has far-reaching consequences beyond just impacting the quality of plastic; it can cause serious health hazards. For instance, PET plastics may contain acetaldehyde, and detergents can contain toxic metals, both of which can be harmful if inhaled or ingested. Eriksen et al. [100] explored the presence of metals in recycled plastic waste and found that metal concentrations increased after the recycling process, although they remained within acceptable limits for food safety [100]. However, the long-term effects of repeated recycling cycles on metal contamination requires further investigation. If metal contamination continues to rise, plastics that undergo numerous reprocessing cycles might no longer meet safety standards.

Furthermore, the economic viability of sorting plastics can be compromised by the high costs associated with the sorting technologies. While these technologies may offer precise sorting capabilities, the expenses involved in implementing and maintaining them can render the process uneconomical. An example is the biodegradability of poly(lactic acid) (PLA), a bio-based plastic often made from corn. Although PLA can be composted or recycled, the market for PLA does not currently justify extensive sorting efforts. As a result, PLA can contaminate high-value and durable plastics, compromising their recyclability.

Despite the ongoing efforts to address the challenges of sorting plastic waste, the continuous development of new additives and polymer mixtures introduces additional complexities to the recycling process. As the plastic industry evolves and new types of plastics emerge, material recovery facilities face the challenge of effectively sorting and processing these novel materials. The dynamic nature of the industry necessitates that sorting technologies adapt and keep pace with these changes to ensure efficient separation and recycling of plastic waste. Failure to effectively sort plastic waste can have detrimental consequences. Even small amounts of contaminants present in the sorted waste stream can lead to cross-contamination of otherwise pure waste streams. This cross-contamination compromises the quality of the recycled material, rendering it less valuable and limiting its potential applications [101]. Furthermore, the presence of contaminants in the recycling process can cause damage to machinery during reprocessing, leading to additional costs and operational disruptions for recycling facilities. Addressing the issue of contamination requires a comprehensive approach. Material recovery facilities need to implement robust sorting systems that can identify and separate different types of plastics accurately, taking into account the evolving landscape of plastic compositions and additives. Additionally, initiatives should focus on improving the infrastructure and logistics of the recycling industry to enable the proper treatment of contaminated recyclables. This includes investing in advanced sorting technologies, enhancing collection and transportation systems, and establishing efficient waste management networks.

### 2.2. Shredding and Extrusion

Shredding is a critical step in reducing the volume of plastic waste and facilitating its processing in mechanical recycling extruders. This process involves breaking down plastic into smaller pieces, making it easier to handle and feed into the extruder. In addition to volume reduction, shredding improves storage and transportation efficiency. Preliminary shredding is often necessary before employing sorting techniques such as flotation. During the shredding process, plastic is fragmented using a circular saw (shredder) or bandsaw. A shredder equipped with rotating blades, driven by an electric motor, is employed for this purpose. The shredded plastic is transformed into flakes measuring between 5 and 10 mm, with smaller sizes resulting in more uniform shapes [102].

From an environmental perspective, grinding plastic waste consumes a significant amount of energy due to the viscoelastic nature of plastic polymers [103]. The energy required for grinding depends on the cuttability of the material. As a result, the different strengths and cuttability of various polymers pose a challenge in achieving a uniform shredding process. Inefficiencies and entanglement can occur during shredding due to the varying mechanical and physicochemical properties of the plastics. Another consideration when recycling plastic films, bags, and sheets is the use of an agglomerator for pre-processing. The agglomerator is a device that consists of a cylinder equipped with several stationary blades and two rotating blades positioned at the base. This configuration induces friction and generates heat. Utilizing agglomeration machines proves to be a highly economical approach to recycle thin-walled polymers. These machines are instrumental in converting loose plastic materials into suitable flakes or chips, which can then be effortlessly fed into an extruder’s hopper for further processing. The agglomerator cuts, preheats, and dries the plastic, increasing its density and quality. Even when the resulting agglomerates or crumbs may not be suitable for direct further processing, they can be mixed with plastic flakes for extrusion [85].

Extrusion is the most widely used method for recycling plastic. In this process, the plastic is blended and fed into an extruder through a hopper. Inside the extruder, the plastic encounters a rotating screw that pushes it into a heated barrel. Gradually, the pressure and heat melt and mix the plastic until it reaches the desired temperature. The molten plastic is then forced through a die, creating a continuous strand or pellet that can be cooled and cut to the desired shape or size. However, concerns arise regarding the mechanical degradation caused by shearing forces and heat during shredding and extrusion. These factors can lead to a reduction in the average molecular weight and mechanical properties of the polymer [104].

Efficient shredding and extrusion processes are crucial for maintaining the quality and properties of recycled plastics. Optimizing these steps can help minimize degradation and ensure the production of high-quality recycled materials. Ongoing research and development efforts focus on improving shredding techniques, enhancing extrusion parameters, and exploring additives or compatibilizers to mitigate the adverse effects of shearing forces and heat. By advancing these technologies, the recycling industry can achieve higher-quality recycled plastics that meet the performance requirements for various applications.

### 2.3. Thermoplastic Blends in Recycling

Polymer blends play a significant role in recycling processes, as they offer the opportunity to create materials with tailored properties by combining different polymers. These blends are physical mixtures of two or more polymers without covalent bonds between them. The interactions between the components can lead to desired property combinations and improved overall performance [105]. Polymer blends are commonly formulated to achieve specific properties or enhance certain aspects of the materials. By combining different polymers, a balance of mechanical strength, flexibility, durability, heat resistance, or chemical resistance can be achieved. However, one challenge that arises in polymer blends is the occurrence of immiscibility and incompatibility. In such cases, the blends may exhibit the presence of large particles from the less abundant component, uneven distribution, and poor adhesion to the surrounding matrix [106]. This challenge is more pronounced in the recycling process, where the original structure of the polymer blend needs to be restored, and the desired arrangement and distribution of the polymer phases must be re-established. Restoring the structure and stabilizing the system during recycling requires appropriate mixing techniques and re-compatibilization. Re-compatibilization involves the addition of chemical additives to improve the interfacial bonding between the polymers [107]. While there are common and readily available compatibilizers, it is important to note that each compatibilizer is designed to address the specific needs of a particular polymer blend. However, considering the vast number of possible polymer blend combinations, the current range of available compatibilizer options is limited from an economic perspective. Developing more cost-effective compatibilizers requires a comprehensive understanding of the interfacial behavior exhibited by different polymers. Such an understanding is crucial for the development of effective and affordable compatibilizers [108]. However, the costs associated with research and experimentation in the development of new compatibilizers pose a challenge. The limited economic feasibility often discourages extensive exploration in this field. Balancing the costs and benefits of developing and implementing compatibilizers is crucial to foster the recycling of polymer blends and maximize their potential in the circular economy of plastics. Ongoing research and innovation in this area can lead to the discovery of cost-effective and efficient compatibilization strategies, enabling the utilization of a broader range of polymer blends in recycling processes.

One of the significant challenges in recycling polymer blends is the potential degradation that the original blend may have undergone, which adds complexity to the recycling process. The degradation behavior of individual polymers within the blend can be influenced by the presence of fillers and other components, further complicating the recycling of polymer blends. Even when compatibilizers are employed, challenges persist, especially in blends containing polymers with different degradation rates. The varying degradation rates among the constituent polymers can impact the overall degradation behavior during recycling [109]. Numerous research studies have focused on investigating the degradation of polymer blends to better understand and address these challenges. For instance, Mistretta et al. [110] demonstrated the positive impact of specific compatibilizers in enhancing the photo-resistance of polymer blends. These studies provide valuable insights into the degradation mechanisms and offer potential strategies for improving the recycling of polymer blends. Continued research and innovation in this area will contribute to advancing the recycling technologies for polymer blends and optimizing their use in the circular economy of plastics.

Research papers have explored various aspects related to polymer blends, extending beyond degradation. For example, Taufiq et al. [111] conducted a study investigating the mechanical properties and morphology of a polymer blend derived from rejected disposable diapers. The study specifically examined the influence of processing temperature on the blend. The results indicated that increasing the compounding temperature led to improved microstructural homogeneity, resulting in enhanced mechanical performance and morphology. In addition to understanding the mechanical properties of polymer blends, researchers have also focused on investigating the impact of recycling on the properties of these blends. One study investigated the reprocessability of high-density polyethylene (HDPE) blends through extrusion molding for multiple cycles [112]. The findings revealed a gradual decline in mechanical performance with each recycling stage, with the most significant drop occurring after the initial recycling step. Another study examined the rheological and mechanical properties of a polymer blend consisting of PLA and PS, prepared using a single screw extruder [113]. The study observed a decrease in the apparent viscosity of the blend with increasing processing cycles, and the flow behavior became more sensitive to shear rate and temperature after recycling. Furthermore, the mechanical properties of the blend deteriorated as the number of processing cycles increased. Although these studies examined different polymer blends, they collectively highlight the negative effects of recycling on the mechanical properties and processability of blends. The decrease in mechanical performance and alterations in rheological behavior after recycling underscore the challenges associated with the recyclability of polymer blends. Consequently, it is essential to develop innovative approaches and technologies that address these challenges, allowing for improved recyclability and the utilization of polymer blends in a sustainable manner.

### 2.4. Degradation of Recycled Plastics

Polymers undergo degradation by various mechanisms throughout their lifecycle and are influenced by factors such as the application environment, operational conditions, and inherent polymer properties. Degradation can occur through processes such as mechanical breakdown, moisture-related damage, heat-induced deterioration, and oxidation due to light exposure. Each degradation mechanism presents unique challenges and can significantly affect the performance and lifespan of polymers. Understanding the diverse ways in which polymers degrade is crucial for optimizing their durability and facilitating effective recycling efforts. During the degradation process, two fundamental mechanisms, chain scission and crosslinking, play a significant role. Polymers consist of interconnected chains of monomers held together by chemical bonds. Chain scission involves the breaking of these bonds within the polymer chain, while crosslinking refers to the formation of chemical bonds that connect different polymer chains. These two mechanisms are in competition with each other, and their prevalence is influenced by factors such as polymer structure, composition, initial molecular weight, and processing temperature [35,114]. By comprehending the mechanisms of polymer degradation, researchers and practitioners can develop strategies to mitigate degradation effects and enhance the recyclability of polymers. This knowledge can guide the selection of suitable recycling processes and conditions that minimize further degradation during recycling. Furthermore, this knowledge aids in the identification of polymers and polymer blends that are more resistant to degradation, thus ensuring the production of high-quality recycled materials. Continued research in understanding and controlling polymer degradation is essential for advancing the circular economy of plastics and promoting sustainable recycling practices.

**Figure 3:**
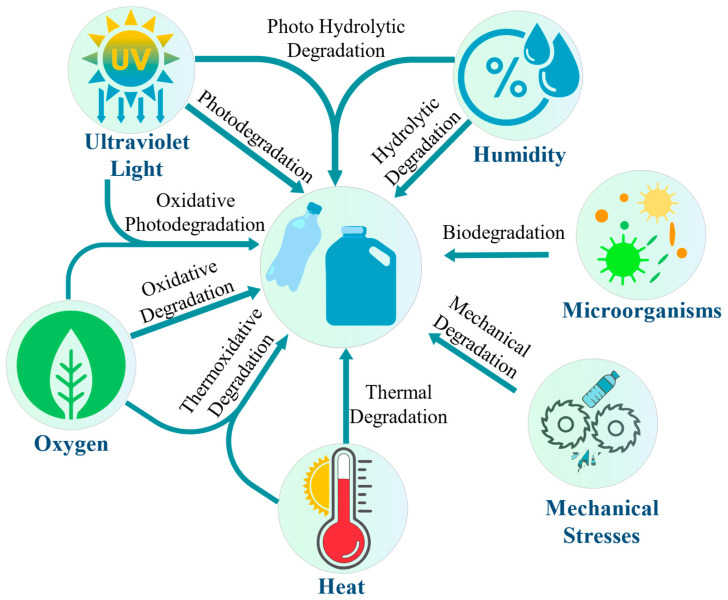
Environmental Factors Leading to Thermoplastic Degradation.

Mechanical degradation is a prominent phenomenon that occurs during the processing of polymers, primarily during shredding and extrusion. These processes subject the polymer to various forms of mechanical stress, which can result in degradation and alterations in its physical and mechanical properties. The intense shear forces exerted on the polymeric chains can lead to their breakdown, causing a decrease in the average molecular weight. This mechanical shear generates significant forces in regions where the polymeric chains are entangled, leading to chain scission [115]. While mechanical forces are not the sole catalysts, they greatly accelerate the degradation process, collaborating with other environmental factors such as temperature, UV radiation, and humidity [116]. Different terms are often used to describe these degradation mechanisms based on their underlying causes. For instance, when temperature and mechanical stress act in combination, this is called thermomechanical degradation. Degradation resulting from UV exposure during processing is known as photodegradation. If oxygen is present alongside UV exposure, it leads to a specific type of degradation called photooxidative degradation. Similarly, degradation caused by moisture during reprocessing is commonly referred to as moisture-induced degradation. Figure 3 depicts the key environmental factors that contribute to material degradation, including heat, UV radiation, humidity, oxygen, and microorganisms. Some of these factors have been discussed earlier. Thermoplastic degradation can occur when any of these factors act independently. However, when two or more of these factors combine, the degradation rate significantly accelerates.

Recognizing the importance of mechanical degradation is essential before delving into the significance of other degradation factors. Extensive research has been conducted to investigate the effects of reprocessing polymers, focusing specifically on the mechanical aspects and the impact of shear forces. These studies aim to understand the behavior of polymers under controlled mechanical stresses while considering other degradation factors. One notable study examined the thermal stability of polystyrene (PS) and revealed its remarkable stability, even at high temperatures of up to 230 degrees Celsius. However, the study highlighted that mechanical stresses encountered during material processing can lead to a reduction in molecular weight, even at lower temperatures. By subjecting the material to similar processing conditions in terms of temperature and duration, but varying the mechanical stresses, distinct variations in molecular weight were observed, which were detected through changes in melt viscosity [117]. This study underscored the influence of mechanical stresses on the degradation of PS, emphasizing the need to carefully consider these factors during processing. Furthermore, a study conducted by Schweighuber et al. [118] provided significant insights into the degradation mechanisms of polyolefins during multiple extrusions using a twin-screw compounder. The investigation revealed that the degradation process is strongly linked to the screw speed employed during extrusion. At lower speeds, chain scission was found to be the predominant degradation mechanism, whereas oxidation processes dominated at higher speeds. These findings highlight the importance of controlling processing conditions to gain a comprehensive understanding of the observed behaviors and their implications for polymer degradation.

As previously mentioned, mechanical degradation alone is not the primary accelerator of polymer degradation. It is the concurrent occurrence of thermal, moisture, or other degradation processes during processing that accelerates the degradation. Moreover, certain degradation factors can amplify the other’s effects, further hastening the degradation process. Thermal degradation plays a significant role in influencing the mechanical properties of processed polymers. When plastics are exposed to higher temperatures during the melting process, thermal degradation can occur, leading to the breaking of the polymeric chain’s weakest bonds and causing a reduction in the compound’s molecular weight [119]. The thermal stability of a polymer is determined by the strength of its weakest bonds [120]. In addition to thermal degradation, the presence of aggressive chemical substances can significantly impact the polymer when exposed to heat. For instance, the interaction between oxygen and the polymer can result in a specific type of degradation known as thermal oxidation [121]. This occurs when oxygen reacts with the polymer, causing further deterioration. Excessive thermal degradation is particularly relevant in the recycling process, especially when dealing with polymer blends. Due to the varying melting temperatures of different plastics, recyclers often encounter challenges in reprocessing plastics based solely on the melting temperature of a single component. As a result, some elements may not melt at all, while others experience excessive thermal degradation.

Numerous research studies have investigated the process of thermal degradation, particularly focusing on the kinetics of thermal and thermo-oxidative degradation in various polymers such as PS, PE, PP, and PET. Peterson et al. [122] conducted a study that revealed the initiation of thermal degradation through random chain scission in the absence of oxygen. However, when heat and oxygen are both present, thermo-oxidative degradation occurs, leading to changes in the degradation characteristics of the polymers. Thermal degradation in PET has also been observed [123,124]. In a recent attempt to prevent degradation, Phanthong et al. introduced an innovative approach that involves attaching a molten resin reservoir unit to extruders. This method facilitates the relaxation and elimination of shear history from the molten state of polymer chains, effectively reducing degradation. As a result, the lamellar structure and morphology can be rejuvenated to closely resemble that of the original virgin materials [125].

During the reprocessing of polymers, moisture is another factor that contributes to degradation and leads to a decrease in molecular weight and durability [126]. Usually, the removal of contaminants and impurities requires mechanical cleaning of the polymers by washing them with water and then drying them. However, insufficient drying may lead to the plastics retaining moisture. Moisture can also be present due to the humidity in the environment. Moisture causes swelling in the amorphous phase, leading to residual hygroscopic stresses and degradation through hydrolysis [127]. The effects of moisture degradation on polycarbonate molding were studied in [128]. Moisture absorption can occur rapidly, with dried pellets exposed to 49% relative humidity at 75 °F reaching the permissible moisture level for molding within 30 min.

The degradation mechanisms discussed earlier primarily focus on factors occurring during processing. However, it is important to recognize that environmental factors such as UV irradiation, moisture, and oxygen also play significant roles in the degradation of polymers. When polymers are exposed to the environment, their degradation occurs gradually over time. Prolonged exposure leads to significant deterioration, a process known as weathering. Weathering is a natural degradation process resulting from the combined effects of UV radiation, atmospheric oxygen, and water. UV radiation, through photophysical and photochemical processes, along with the presence of oxygen and water, causes oxidative and hydrolytic effects on the polymers [129]. Oxygen reacts with polymer chains, forming oxygenated groups in a phenomenon known as photo-oxidation, resulting in the gradual degradation of polymers [130]. Additionally, water absorption by synthetic materials and coatings from humidity and wetness can lead to hydration of surface layers and mechanical stress on the dry subsurface layers. Simultaneously, UV rays from sunlight can accelerate the degradation process by initiating photo-degradation, breaking down chemical bonds in the polymer material and leading to a gradual loss of physical properties [131]. Numerous research papers have examined the impact of UV aging on the mechanical and fracture characteristics of thermoplastic polymers.

The impact of degradation on the mechanical recycling process is evident, and the quality of the waste material before recycling plays a crucial role in determining the overall success of the process. While the direct influence of polymer degradation on technical stages such as sorting or cleaning remains inconclusive, chemically degraded waste materials require stricter controls and greater precision to achieve the desired results. Handling lower quality waste materials necessitates heightened attention and control throughout the recycling process.

In summary, environmental factors such as UV irradiation, moisture, and oxygen contribute to the degradation of polymers in the environment, leading to weathering and gradual deterioration. UV radiation causes photo-oxidation and initiates photo-degradation, while moisture absorption and exposure to oxygen contribute to hydrolytic and oxidative effects on the polymers. Research studies have highlighted the impact of UV aging on the mechanical properties of polymers, revealing the competition between chain scission and crosslinking mechanisms. Understanding the degradation caused by environmental factors is essential in managing the quality of waste materials and implementing effective recycling processes. By considering the effects of degradation, greater attention can be given to controlling and improving the recycling of lower quality waste materials.

## 3. Integrating Plastics into a Circular Economy through the 3D Printing Process

Addressing the urgency of global challenges requires a transition from lifestyles solely centered on consumption, towards more sustainable activities. The shift is driven by the aim of meeting the present needs, while safeguarding the well-being of future generations. To achieve sustainability, it is crucial to seek a balance between environmental, social, and economic considerations [132]. Environmental sustainability involves protecting natural resources, reducing carbon emissions, and minimizing pollution. This requires adopting renewable energy, sustainable land management approaches, and eco-friendly practices across industries. Social sustainability focuses on promoting social justice, equity, and inclusion [133]. Economic sustainability involves maintaining a thriving economy while ensuring it is not at the expense of the environment or society. It involves creating economic systems that promote sustainable development, job creation, and a fair distribution of wealth and resources [134]. Achieving sustainability requires collaboration among governments, businesses, and individuals to develop and implement sustainable policies, technologies, and practices [135]. This includes investing in renewable energy, sustainable agriculture, eco-friendly transportation, and circular economy principles to achieve a more sustainable future [136,137].

The concept of a circular economy is important for achieving sustainability, as it focuses on reducing waste and maximizing resource efficiency, leading to a more robust and sustainable future [138]. A circular economy model aims to keep materials, products, and waste in use for as long as possible through reuse, repair, and recycling. The objective is to minimize waste and its environmental impact by reducing the demand for virgin materials and lowering energy consumption. This model replaces the linear economy approach known as the “take-make-dispose” model [134,139]. The linear economy relies on unlimited resources to produce, use, and dispose of products, which is unsustainable and disregards the negative environmental consequences and the finite nature of natural resources [140]. To transition to a circular economy, industries must adopt strategies for extending the life of products and materials, creating a sustainable and resilient circular economy. Recycling plastic within the circular economy is a solution for sustainability as it promotes resource efficiency and waste reduction. Plastic waste poses a significant environmental threat due to its slow biodegradability and persistent presence in the ecosystem [141]. A circular economy aims to extend the useful life of plastic through the utilization of various frameworks, such as the 6 Rs or 9 Rs [142].

**Figure 4:**
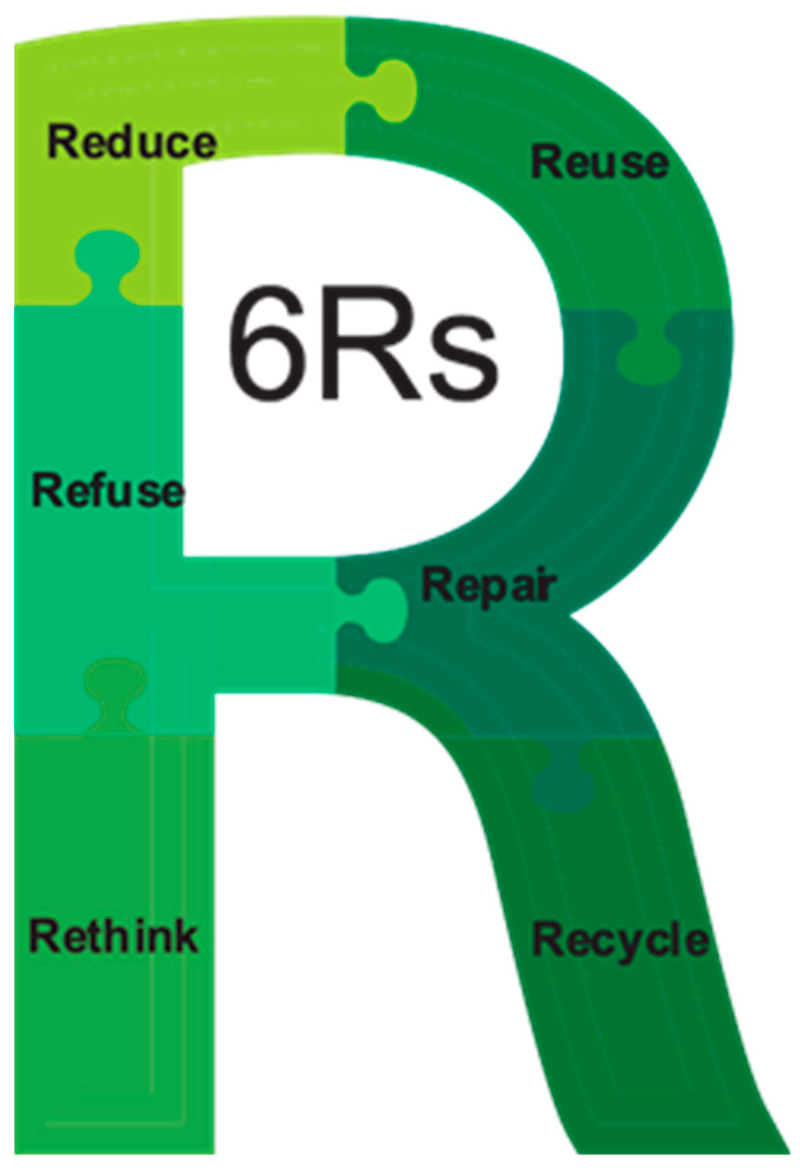
Six Rs of Sustainability.

The sustainability framework, developed by multiple industries, provides an outline for waste reduction and the promotion of sustainable practices. The 6 Rs framework shown in Figure 4 includes Reduce, Reuse, Recycle, Repair, Refuse, and Rethink, while the 9 Rs framework expands to include Rs such as Refurbish, Remanufacture, Repurpose, or Recover [142,143,144]. While the first three Rs are widely known and promoted, all the Rs are important for achieving a sustainable future. Repairing and reusing products extends their lifespan and reduces the need for new materials and energy in manufacturing. Refusing single-use products is a critical step for filtering out items that harm the environment. By incorporating all the Rs into the circular economy, society can significantly reduce its environmental impact and promote sustainable practices for future generations [145]. Replacing single-use plastics with environmentally friendly alternatives, such as reusable metal or bamboo straws and refillable glass or stainless-steel water bottles, can significantly reduce waste [146]. Governments and businesses are implementing policies and offering incentives to encourage the use of reusable alternatives to single-use plastics, fostering a shift towards sustainability [147,148]. Integrating plastic recycling into the circular economy is vital for reducing plastic waste, conserving resources, and promoting sustainable practices [147]. These efforts aim to close the loop on plastic waste reduction within the circular economy.

Establishing plastic recycling as part of the circular economy requires the creation of a closed-loop system that prioritizes waste management frameworks. It begins with product design in which designers prioritize recyclability by using fewer complex parts and a higher percentage of the same plastic to enhance recycling efficiency and effectiveness [149]. Establishing a supply chain that prioritizes recycling is also crucial for a circular economy of plastics. This involves partnerships with waste management companies and specialized recycling facilities capable of handling different types of plastic, as well as localized platforms to facilitate recycling and eliminate the need for long-distance transportation to centralized recycling facilities [150]. Policymakers and businesses must prioritize sustainability concerns and integrate circular economy principles into their strategies to support the shift towards a circular economy for plastic [151]. By integrating these strategies, it is possible to reduce waste, conserve resources, mitigate the negative impacts of plastic production and disposal on the environment and human health, and gain a competitive advantage in the marketplace through improved efficiency and reduced costs [142]. As described in Section 1, thermoplastics, given their widespread use in various applications, are a major focus for recycling efforts [32].

### 3.1. Recycling of Thermoplastics

Thermoplastic polymers are widely used in various products, such as packaging, consumer goods, and industrial applications, due to their unique properties, including durability, flexibility, and relative resistance to degradation [152]. However, the linear model of polymer consumption and disposal has resulted in significant environmental challenges [32]. According to the report “What a Waste 2.0” [15], the world generated 242 million tons of plastic waste in 2016, and this figure is predicted to increase to 460 million tons per year by 2030 if the current trend continues. The accumulation of plastic waste and its disposal in landfills or incineration contribute to environmental pollution and greenhouse gas emissions, exacerbating climate change [153]. To address this issue, it is crucial to integrate polymers into the circular economy, which involves reducing production and consumption, promoting reuse, recycling, and recovery cycles [154]. In the context of mechanical recycling, significant efforts have been made to recycle thermoplastics using injection molding, thermoforming, and 3D printing technologies as described in the next three subsections.

#### 3.1.1. Recycling Thermoplastics through Injection Molding

Injection molding is a manufacturing method for producing a wide range of plastic products, including those made from recycled materials [155]. The process of recycling by injection molding typically involves a series of steps including collection, sorting, cleaning, and size reduction to create plastic pellets. These pellets are then dried to remove moisture as it affects the production process. Next, pellets are melted and injected into a mold, where they form the desired product [156]. However, there are several advantages to using injection molding with recycled plastic. Firstly, it is a cost-effective solution for plastic recycling, as shredded recycled plastic makes a pellet form feedstock to the injection molding and the process can be repeated multiple times. Additionally, injection molding reduces waste by reusing plastic that would otherwise end up in landfills or oceans [157]. The flexibility of injection molding enables the production of complex shapes and sizes, making it suitable for a wide range of plastic products. Through the utilization of recycled plastic and injection molding, this approach has the potential to make a significant difference in waste reduction and promote a more sustainable environment [158].

An example of plastic recycling involves the use of recycled polyethylene terephthalate (rPET) in injection-molded products, sourced from post-consumer PET bottle recycling. This practice not only reduces plastic waste, but also lowers raw material costs. Studies have explored various additives, processing parameters, and types of rPET waste to enhance the properties of injection-molded rPET, demonstrating the potential for sustainable solutions and value-added product creation in addressing the plastic waste issue [159]. Another effort in injection molding has been extended to plastic waste collected from marine environments. Utilizing different manufacturing technologies, including injection molding, 3D printing, and thermoforming, researchers have examined the recyclability of PET, HDPE, and PP plastic waste retrieved from the ocean. Comparative analysis with virgin plastics indicates that recycled plastic products exhibit comparable properties, highlighting the potential for effective recycling of marine plastic waste and the significance of improved waste management strategies in marine environments [160]. Thermoforming represents another method employed in the recycling of thermoplastics and is covered in the next subsection.

#### 3.1.2. Recycling Thermoplastic through Thermoforming

Thermoforming is a manufacturing process used in plastic recycling. The technique starts by heating a plastic sheet to its softening point, stretching it over a mold, and then vacuum-forming it into the desired shape. This method has found extensive use in recycling applications, facilitating the creation of diverse products from recycled plastic, such as food packaging, automotive parts, and consumer goods. One noteworthy application of thermoforming is its role in producing high-stiffness trays. The tray’s inherent structural integrity and rigidity makes it ideal for tasks demanding stability and resistance to deformation. A key advantage lies in the ability to carefully select appropriate materials and optimize the thermoforming process, thereby yielding trays with superior stiffness tailored to meet specific requirements [161].

While injection molding and thermoforming are commonly used technologies in plastic recycling, additive manufacturing through 3D printing provides an innovative approach. Unlike injection molding and thermoforming, which involve heating and molding plastic into predetermined shapes, 3D printing allows for the creation of complex and customizable objects using recycled plastic materials [156]. Therefore, this section will explore 3D printing as a novel approach to plastic recycling.

### 3.2. Recycling of Thermoplastics through Additive Manufacturing: Opportunities and Challenges

**Figure 5:**
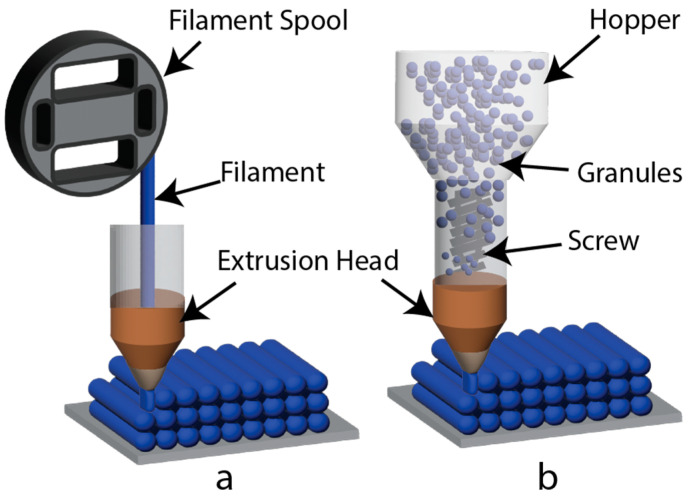
Illustration of Additive Manufacturing Techniques: (**a**) Fused Filament Fabrication and (**b**) Fused Granule Fabrication.

Additive manufacturing, commonly known as 3D printing, presents a promising solution for integrating plastics into the circular economy and recycling plastic waste [162]. This technology allows for the creation of complex objects while optimizing material use and reducing waste during the manufacturing process [21]. One of the widely used additive manufacturing processes is fused filament fabrication (FFF), in which polymer material is deposited layer by layer over a heated bed, as depicted in Figure 5a. This approach results in efficient material utilization and minimizes waste generation. Another technology employed in additive manufacturing is fused granule fabrication (FGF). In this method, illustrated in Figure 5b, feedstock for 3D printing is provided in the form of granules. The granules are heated and melted by passing through a screw extruder, after which the molten material is extruded through an extrusion nozzle to build the desired object [163]. FGF offers an alternative approach to incorporate recycled polymers into the additive manufacturing process, promoting the reuse of plastic waste and contributing to a more sustainable production cycle. By harnessing the potential of 3D printing, the circular economy can be further advanced, leading to reduced material wastage and improved environmental impact.

The utilization of additive manufacturing and 3D printing technology in recycling polymers presents numerous environmental benefits. It enables the transformation of plastic waste into new products, reducing the amount of waste generated and the reliance on raw materials, energy, and water typically required in traditional manufacturing processes [164]. Recycling plastic materials through 3D printing can significantly reduce waste, diverting plastic from landfills. It also helps conserve resources by reusing plastic, reducing the need for new plastic production and the associated energy consumption [165]. The recycling process itself typically requires less energy and a reduced carbon footprint compared to producing new plastic from raw materials. Furthermore, recycling can create new markets for recycled plastic materials, promoting economic diversification and generating new business opportunities [166]. Moreover, 3D printing allows for the creation of customized and complex designs as well as minimizing material waste during production [167]. While additive manufacturing offers valuable advantages, it also presents challenges that need to be addressed.

Effectively recycling polymers for 3D printing presents a significant challenge, requiring a thorough understanding of which materials can be recycled without compromising mechanical properties [54]. Based on the reviewed literature, research studies have explored the recyclability of various polymers, with a focus on acrylonitrile butadiene styrene (ABS), polylactic acid (PLA), polyethylene terephthalate (PET), and polypropylene (PP). However, fewer studies have investigated the recycling potential of polystyrene (PS), high-density polyethylene (HDPE), and low-density polyethylene (LDPE) polymers [168]. To address this challenge, researchers are dedicated to comprehending the mechanical properties of recycled materials and developing strategies to mitigate degradation. Techniques such as incorporating additives or blending multiple polymers to enhance filament flexibility will be further discussed in Section 4 [169,170].

A significant challenge in thermoplastic recycling in general and for 3D printing in particular is the collection and sorting of plastic materials, as discussed in Section 2.1. Ensuring the proper sorting of plastics becomes critical due to the variation in melting points among different polymers. This discrepancy can present a major difficulty during the 3D printing process. Proper sorting of plastics becomes a crucial step that greatly influences the success of 3D printing with recycled materials [171].

It is widely acknowledged that not all materials are suitable for recycling by 3D printing. Consequently, various methods were explored involving specialized equipment to address the challenges associated with printing these materials. It was often concluded that recycling such materials may not be feasible or would be financially irrelevant. For example, let’s examine the recycling of composite materials commonly used in packaging, which consist of both polymers and aluminum [172]. While it is technically possible to recycle these materials, the process demands substantial resources for effective processing. Furthermore, some of these difficult-to-recycle plastics can present safety and regulatory concerns, particularly if they release hazardous fumes or byproducts during the recycling and printing processes [173]. Hence, it is important to approach these materials with caution and implement appropriate mitigation strategies.

Ensuring that recycled plastics meet the required specifications for 3D printing can be difficult. Moreover, recycled thermoplastic materials may exhibit different physical characteristics compared to virgin materials, potentially influencing the performance of the printed object. Variations in melting points, viscosities, and flow characteristics can affect printability, leading to issues such as warping, poor layer adhesion, and surface finish problems [174]. This can lead to issues such as inadequate layer adhesion, stringing, and clogging, which can compromise the quality of the print [175]. Furthermore, achieving proper adhesion of the material to the print bed can be challenging, especially when using recycled polymer materials that may have different surface qualities compared to virgin materials. This can result in problems such as warping and poor layer adhesion, affecting the overall print quality. Additionally, factors such as print speed, layer height, and cooling settings can have a significant impact on printing quality and may contribute to failed prints [176], which will be discussed in Section 3.4.

When considering the use of recycled plastic materials, the factor of cost plays a crucial role. While it has been argued that recycled plastics can be more expensive than their virgin counterparts due to additional processing and quality control requirements [2], a deeper examination of this cost dynamic is essential. Choosing between virgin and recycled plastic involves a complex decision-making process that extends beyond mere cost considerations. Recycled plastic undoubtedly offers substantial environmental benefits in terms of sustainability, but it may entail additional processing stages, leading to increased expenses. The higher cost associated with recycled plastics typically stems from various stages, including collection, sorting, cleaning, and transportation. However, the adoption of a distributed recycling approach can help offset these added costs by eliminating the need for transportation to centralized recycling facilities. Moreover, it is important to note that the cost of the raw material itself is effectively eliminated during the recycling process. This holistic perspective underscores that the choice between virgin and recycled plastics involves a nuanced evaluation of multiple factors beyond cost alone.

Nonetheless, it is imperative for the recycling community to acknowledge the multifaceted benefits that recycling offers, extending beyond its environmental advantages to encompass economic gains. In order to establish a tangible benchmark for quantifying the costs associated with the implementation of a DRAM approach, a meticulous estimation has been undertaken. This estimation stands as a foundational point for comparison, particularly against commercially available recycled PET (rPET) sourced from re3D, Houston, TX, USA. The principal objective of this model is to foster community engagement, with a significant portion of the endeavor relying on the dedicated efforts of volunteers. Additionally, the intent is to manufacture pellets from plastic products collected within the community, procured at no cost. However, there exist other essential cost considerations in the process. These encompass various expenditures, including water bills associated with cleaning and electricity bills for operations such as shredding and drying. To provide a comprehensive overview, Table 3 outlines the projected expenses associated with the production of 1 kg of PET using conventional equipment.

The initial estimate points suggest significant cost savings for the community when compared to the price of rPET, which stands at $19.64 per kilogram. Consistent with our calculations, Alexandre et al., have indicated that the production costs of fused granular fabrication pellets can be efficiently reduced to less than $1 per kilogram of material [177]. While we acknowledge the potential presence of supplementary costs, notably labor and equipment expenses, it is imperative to assess the broader financial implications. An optimally staffed recycling center would ideally involve three to five skilled individuals responsible for sorting, cleaning, and shredding operations, complemented by one individual overseeing the collection and the team. Our data were collected using equipment available in our laboratory, a Filabot Reclaimer (Filabot, Barre, Vermont, USA), valued at $8190, and the drying ovens, akin to the Thermo Scientific 3488M-1 Imperial V model, ranged in price from $2000 to $5000. Additionally, prudent financial planning should encompass an annual maintenance cost, equivalent to 1–3% of the machine’s assessed value, to ensure sustained operational efficiency and longevity.

In devising a simplified model for these recycling centers, let’s assume each center is equipped with a single industrial shredding machine capable of processing 50 kg of plastic material in an 8-h shift. This limitation would mean that each center could handle up to 50 kg of material every 8 h, constrained by the shredding process. In the United States, the average per capita daily plastic consumption stands at approximately 0.605 kg/day. Based on this, a single shredder can effectively serve and process materials from around 83 recyclers. Now, let’s consider that roughly 20% of the community members are inclined towards distributed recycling. In such a scenario, each center would need to meet the daily demands of a population comprising approximately 415 individuals within its vicinity. Applying this model to Michigan, it becomes apparent that recycling centers would need to be strategically located within an approximate radius of 2.8 km to efficiently serve the community. For instance, in Saginaw County, this would necessitate the establishment of approximately 24 recycling centers to accommodate the local population’s recycling needs. Larger communities may require additional equipment and centers for effective recycling management.

However, in the context of additive manufacturing, the fused granule fabrication (FGF) process offers a more direct route for using recycled materials [178]. Instead of creating a spool of filament for the fused filament fabrication (FFF) process, which requires additional steps in processing, the FGF process allows the utilization of recycled material promptly after chopping and cleaning. This streamlined approach not only simplifies the production process but also has the potential to lower overall costs, making recycled plastics a viable and sustainable option for additive manufacturing.

Overall, FFF and FGF provide a promising pathway for recycling thermoplastics and integrating polymers into the circular economy. By addressing the challenges associated with polymer recycling, such as material selection, printability optimization, and cost considerations, the environmental impact of plastic production and disposal can be significantly reduced. Table 4 highlights additional initiatives complementing those discussed in Section 3.1, with a focus on injection molding, thermoforming, and 3D printing processes. These ongoing efforts are dedicated to further advancing the recycling of plastic materials, demonstrating the versatility and potential of various recycling techniques in creating sustainable solutions.

### 3.3. Pathway to Community-Scale Recycling through Additive Manufacturing

Over the past decade, the concept of distributed recycling by additive manufacturing (DRAM) has garnered considerable attention in academic literature [200]. DRAM has been explored not only as an individual or household practice but also as a viable community-based solution [162]. Individual DRAM practice demands substantial skill, effort, and capital, so it may result in low machine utilization. Therefore, our focus lies on the broader implementation of DRAM at the community scale, where it can be adopted by small enterprises, non-profits, or volunteer initiatives. In this review, we outline the essential steps for establishing DRAM at the community level, paving the way for a more accessible and sustainable approach to recycling and additive manufacturing.

**Figure 6:**
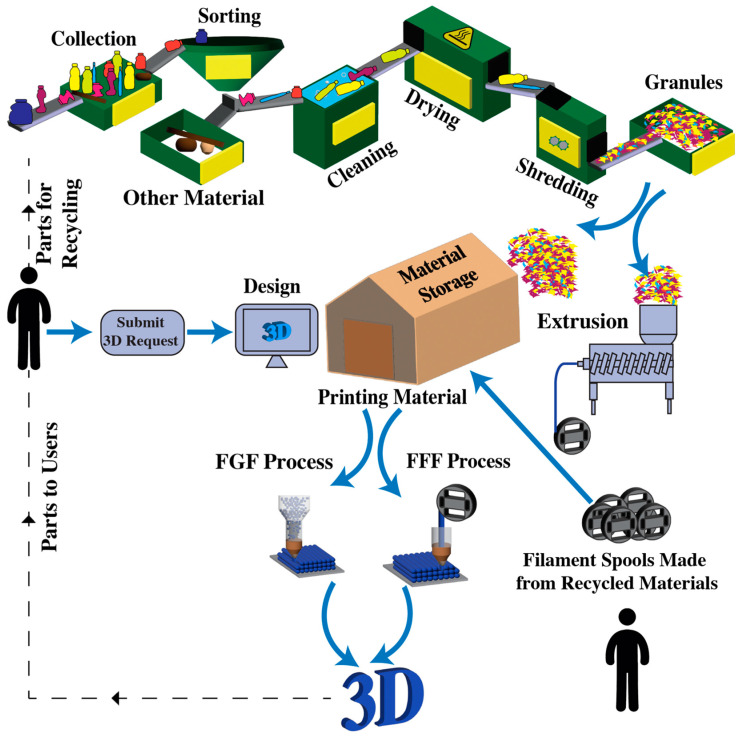
Transformation of plastic waste into a 3D printing feedstock through a local recycling process.

**Figure 7:**
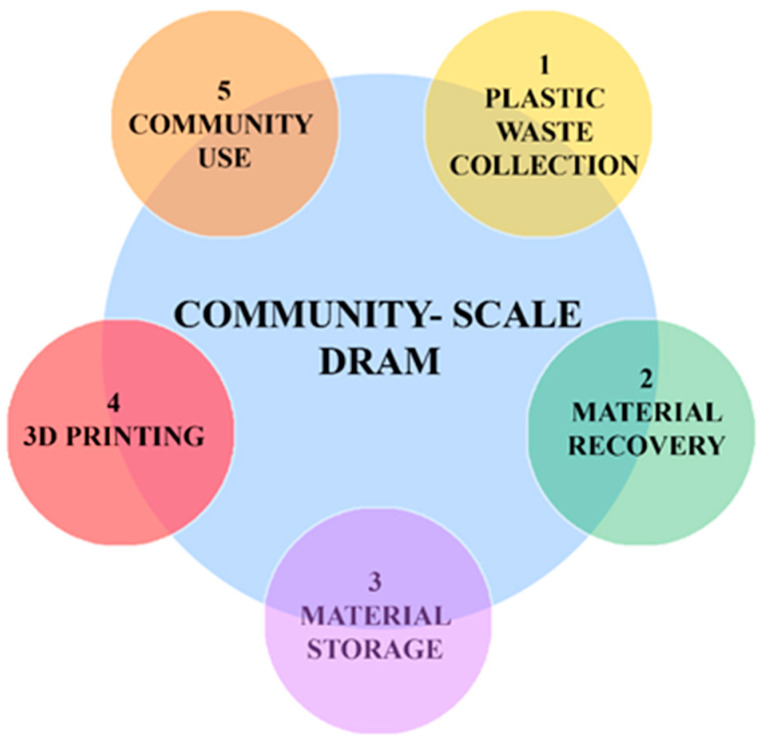
Stages of Distributed Recycling for 3D Printing: From Waste Collection to Community Use.

To prepare plastic waste for 3D printing, a series of essential steps are undertaken to transform discarded materials into usable feedstock. The process commences with the collection and sorting of plastic waste, followed by thorough cleaning and shredding into smaller pieces. Subsequently, the shredded plastic undergoes extrusion or other processing techniques to create filament or pellets suitable for 3D printing, as depicted in Figure 6. This transformation is a key factor in converting plastic waste into a valuable resource, promoting sustainability and circularity by reducing environmental impact and maximizing material reuse. Advocating a decentralized approach to waste recycling, our review paper proposes the concept of distributed recycling within the community. This approach eliminates the need for transportation to a centralized recycling center, promoting greater efficiency and reduced logistics. Consequently, our review focuses on the process of establishing a distributed recycling unit consisting of five stages, as depicted in Figure 7. The five stages are: plastic waste collection, material recovery, material storage, 3D printing, and community use. By highlighting these stages, our review aims to promote the implementation of an effective distributed recycling unit, fostering community involvement and contributing to a more sustainable and responsible waste management system.

*Pathway to Decentralized Recycling*

Sorting waste and ensuring its proper recycling has always been a challenge due to contamination issues [29,188]. However, communities can overcome this challenge by establishing efficient sorting systems and educating residents on proper recycling practices. Initiating a recycling program, as illustrated in Figure 6, allows communities to determine the procedures for sorting and collection while developing clear policies on what can be recycled. To involve the local community in the waste collection process, collection containers can be placed in public spaces, schools, and at community events [201]. Additionally, setting up drop-off locations enables community members to contribute by bringing their already recycled plastic.

Once the plastic waste is collected, the next crucial step is shredding and preparing the recycled plastic for 3D printing. Mechanical shredders are used to downsize the plastic into desired sizes, typically flakes or granules. Proper sizing is essential, whether producing filaments or directly 3D printing using a fused granule fabrication (FGF) 3D printer. Community-scale recycling centers can benefit from cost-effective and accessible shredding systems, such as the open-source plastic granulator, which can be built by members of the local community with expertise in mechanical and electrical fabrication [202]. Alternatively, the concept of the “Green Fab Lab” can be adopted, focusing on establishing local recycling facilities [203].

Before 3D printing, all recycled materials must be thoroughly cleaned and dried to avoid moisture-related issues during the printing process. Common methods for drying include air and vacuum drying at temperatures below the glass transition temperature [204]. Communities can invest in a drying oven or dehumidifier to ensure proper drying of materials. By implementing community-based waste recycling and 3D printing, communities can take significant steps towards sustainability, reducing environmental impact, and promoting the circular economy. Through collective efforts, waste can be transformed into valuable resources, fostering a more sustainable and eco-friendly future for generations to come.

*The Process of Filament Fabrication*

In the recycling and 3D printing process, as depicted in Figure 6, the dried and stored plastic material undergoes extrusion to create filament suitable for 3D printing. In the reviewed literature, the most common filament diameter used for 3D printers is 1.75 mm, with tolerance levels ranging from +/− 0.04 [194] to 0.1 mm [192]. Maintaining the filament diameter within an acceptable range of tolerance is critical as it directly impacts the printing quality and the outcome of the print. To ensure smooth and consistent extrusion during printing, it is essential for the filament diameter to be consistent throughout the spool. Any variations in diameter can lead to issues such as clogging or under-extrusion, compromising the quality of the 3D printed object. Therefore, stringent quality control measures should be in place during filament fabrication to guarantee uniformity in diameter. Therefore, [193] used a laser micrometer with an accuracy of ±2 μm to monitor the filament diameter during extrusion. Moreover, the filament must be free of contaminants such as dust and particles [205]. Contaminants can lead to clogging in the 3D printer nozzle or poor adhesion between layers, resulting in flawed prints. Properly cleaning and filtering the recycled material before extrusion is crucial to eliminate any potential contaminants. A mesh filter is often used inside the extruder nozzle as well.

*Direct Printing Using Granules*

An alternative method for 3D printing with recycled polymers involves the direct use of granules as feedstock, eliminating the need for intermediate steps such as filament production. In this approach, plastic waste is processed into granules of a specific size and then directly fed into a 3D printer equipped with a hopper, bypassing the step of filament production. Table 5 is an example of printing parameters using an open-source 3D printer Gigabot X 2 XLT (re3d, Texas, USA) to print a recycled polymer (PLA) [206]. While direct printing using granules shows potential for sustainable 3D printing, it may require modifications to the 3D printer to prevent clogging of granules in the hopper. Advancements in open-source technology and the availability of pelletizer choppers or open-source granulators offer feasible solutions for the local community to create granules of the appropriate size without the need to produce filament for 3D printers [207].

### 3.4. Controlling the Printing Quality of the Recycling Plastic

**Figure 8:**
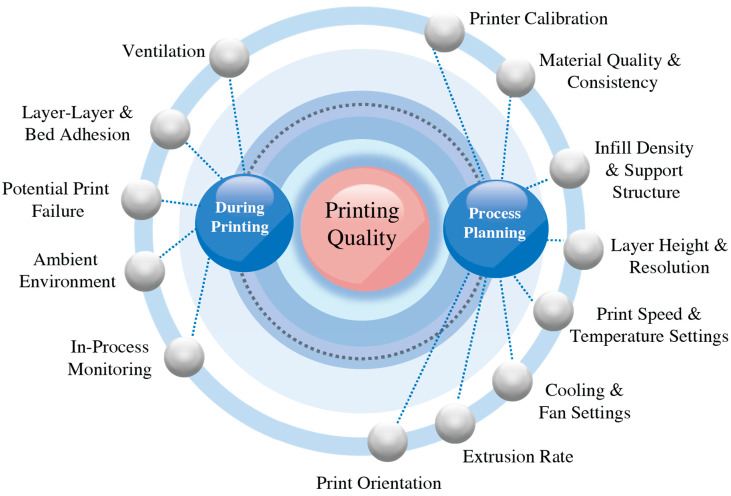
Printing Quality Control for 3D Printing.

To ensure accurate and reliable 3D printing of the recycled plastic, process control is crucial. With the growing popularity of 3D printing technology, understanding and optimizing printing process parameters is key. The process control aspects of FFF and FGF 3D printing encompass various factors, including printing material selection, printing temperature, layer thickness, and print speed. Wu and Chen conducted an analysis using cause-and-effect analysis [208] to identify factors contributing to poor print quality, which are summarized in Figure 8. By monitoring and controlling these factors, it is possible to reduce failed prints and improve overall printing performance. These factors are divided into two main categories: before printing (process planning) and during printing.

#### 3.4.1. Process Planning

Several important 3D printing parameters have a big impact on the success of the printing process. Figure 8 illustrates the important elements of control that must be considered during the process planning phase. Layer height, printing speed, extrusion rate, and temperature settings are important factors in 3D printing [209]. Lower layer heights produce finer vertical details but lengthen print time. Additionally, the material’s adhesion to the prior layer depends on the extrusion rate and temperature. Therefore, consistent print quality is ensured by using correct and optimized settings [210].

Other factors that affect print quality are settings in software used for slicing a 3D design into machine instructions for printing. These important slicer settings include support settings, infill density, and print orientation. The creation of support structures that stabilize overhanging components while printing is controlled by support material parameters. Better support for complex geometries is ensured by higher support density, but it also requires more material and post-processing work. The infill density parameter determines how much material is used to fill the inside of the part, which affects the print’s strength and weight. Greater structural integrity is achieved with higher infill densities, but at the cost of increased printing time and material usage. Another factor that needs to be considered is the print orientation, which affects the object’s structural integrity and surface finish [210,211].

#### 3.4.2. Control during Printing

Once the printing instructions are sent to the 3D printer, the print begins. Most of the print monitoring tasks are left to the human operator, with typical printers using closed loop control of only heated bed and extruder temperatures. The operator must monitor the bed adhesion and layer-to-layer adhesion to detect failures that require nozzle or bed cleaning and a material reset. Additionally, print chamber air temperature plays an important role in cooling and thus part shrinkage and residual stress-driven warpage. The chamber temperature requirements vary based on the specific plastic being printed. In particular, PLA requires an open printer enclosure for rapid cooling below its approximately 60 °C glass transition temperature, while other plastics demand a closed chamber or even a heated chamber for optimal results. Moreover, controlling the ambient humidity is also important to ensure the print quality and success [212].

#### 3.4.3. In-Process Monitoring

Nevertheless, advancements are continuously being made to enhance the process control of 3D printing. In-process monitoring systems incorporating sensors and machine learning algorithms have emerged as a modern technique. These systems provide real-time feedback and enable modification of process parameters during printing. A study has proposed the use of sensors and machine learning algorithms to prevent nozzle clogging, achieving an impressive prediction accuracy rate of 97.2% for the future state of the 3D print [213]. Such systems aim to eliminate the need for operator monitoring and enhance print success. Based on the specific requirements of the application, the community can develop a similar system tailored to their needs, further improving the process control of 3D printing within a community recycling center and enhancing the reliability and efficiency of their printing operations [214].

#### 3.4.4. Empowering Local Communities through Self-Sustaining Recycling Centers

Extrapolating the idea of DRAM to the context of a local community, we envision the concept of a self-sustaining recycling center in the local community practicing collection, sorting, cleaning, processing, and 3D printing of plastics for applications in the local community. Several essential pieces to this center are the trained operators, the machines, the education and engagement of potential customers or users of the parts to be produced by DRAM. This idea is similar to the Precious Plastics initiative for local centers to collect and recycle plastics and then use the manufacturing processes of die extrusion, injection molding, and thermoforming. To those processes, we add additive manufacturing processes. Precious Plastics fosters the sustainment of such local centers with participating machine shops and marketplaces distributed regionally and with knowledge sharing online. By taking ownership of the recycling process and developing expertise within the community, the center becomes more self-reliant and fosters local economic growth and learning. With trained operators, the proposed DRAM center can operate efficiently, producing high-quality recycled materials and products through 3D printing. This not only promotes environmental sustainability by reducing plastic waste but also encourages active community participation in recycling initiatives. By involving the local community in the process, 3D printing with recycled materials becomes a collaborative effort that fosters environmental consciousness and community engagement. As a result, the community benefits from a self-sustaining recycling center that contributes to a cleaner and more sustainable environment while promoting economic and social growth within the community itself.

In the context of a local self-sustaining recycling center, the application of 3D printing technology offers a multipurpose and sustainable approach to addressing various household needs and the promotion of community empowerment. Through 3D printing, communities can produce many household items that might eliminate the need to purchase from the market, ranging from replacement parts such as vacuum attachments, gardening tools, home accessories and many others. This approach encourages the utilization of the resources within the local community, which can help in community engagement and innovation. Such combined efforts align with the vision of sustainability and circular economy, contributing to a more self-reliant community model.

## 4. Mechanical Properties of Recycled Polymers

The successful integration of plastics into a circular economy requires maintaining the mechanical properties of recycled thermoplastic structures to a high standard. This emphasis ensures that the recycled materials retain their strength and reliability, enabling them to endure repeated use without any compromise in performance. By prioritizing the quality of recycled thermoplastics, it is possible to create a closed-loop system in which materials are continuously reused, minimizing waste and reducing the demand for virgin resources. To achieve this objective, it is essential to understand the mechanical behavior of thermoplastics before and after recycling and to uncover the underlying mechanisms involved in their property changes. Polymers are composed of large chain molecules, with carbon atoms forming the backbone of the chain. Polymerization is the process of linking monomers, the building blocks of polymers, to create these chains. Thermoplastic polymers are characterized by linear chains that are connected to side groups, and in some cases, these chains may have branches, forming branched structures [215]. This unique feature distinguishes thermoplastics from other polymers because their chains are not crosslinked. As a result, they can be easily melted at relatively low temperatures, which contributes to their excellent recyclability potential. Furthermore, the arrangement of these linear chains can vary, and these structural characteristics significantly affect the mechanical behavior and performance of the polymer under different conditions. Many thermoplastics display a semicrystalline morphology, with a molecular structure comprising amorphous regions of randomly oriented molecular chains and crystalline regions that exhibit a highly ordered and repetitive chain arrangement with hydrogen bonding between chains. However, it is essential to note that thermoplastics can also exist in an entirely amorphous state such as polystyrene (PS), polycarbonate (PC), and polymethyl methacrylate (PMMA), also known as acrylic. The mechanisms of deformation as well as mechanisms of degradation are tightly linked to the macromolecular structure of the thermoplastic.

**Figure 9:**
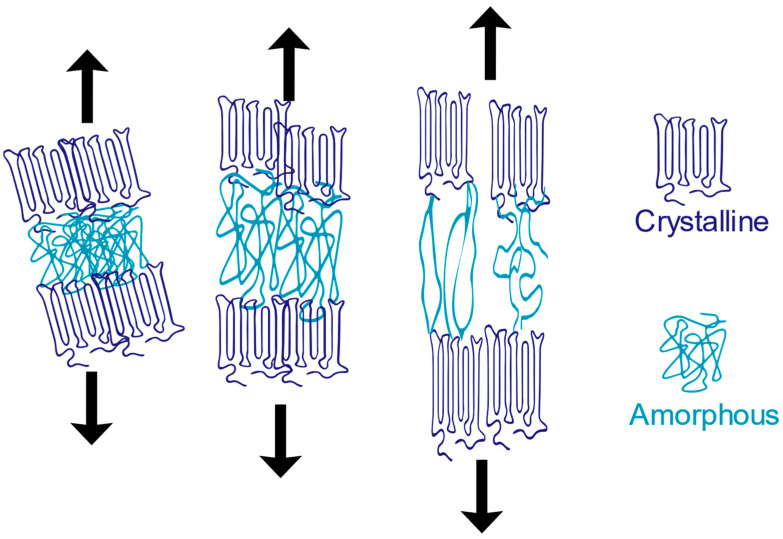
Microstructural Evolution under Deformation in a Semicrystalline Polymer.

The amorphous thermoplastics featuring a network of randomly organized and entangled polymer chains [216], exhibit an isotropic behavior. When a semicrystalline thermoplastic is subjected to mechanical forces, the amorphous phase experiences initial elongation, leading to the stretching and unraveling of some polymer chains, and the breaking of weaker intermolecular bonds [216]. As the deformation progresses, the load is primarily borne by the crystalline region of the thermoplastic. This causes the crystalline lamellae to align in the direction of the first principal stress. This point coincides with the macroscopic plastic yielding, characterized by localized narrowing and uneven deformation, leading to a concentrated area of strain [217]. With further applied force, the crystalline phase starts to separate into distinct crystalline blocks [218]. As the deformation continues, both the crystalline blocks and the chains of the amorphous region that connect them become highly elongated, resulting in the formation of a fibrillar-like structure. Both these crystalline block segments and the interlinking amorphous regions adjust their orientation to align with the direction of maximum stretch, known as the principal stretch direction [219]. However, this hardening is constrained by the macromolecular chains’ ability to stretch. Once their extensibility limit is reached, fracture occurs. This intricate fibrillar arrangement contributes to the thermoplastic’s ability to withstand deformation and maintain its mechanical integrity under stress. Figure 9 illustrates the described deformation mechanisms occurring within the crystalline and amorphous regions associated with the semicrystalline polymer’s mechanical response. The interplay between the amorphous and crystalline regions within thermoplastics enables a complex mechanical response to external forces. The amorphous phase provides resilience and flexibility, allowing the material to absorb impacts and recover its shape, while the crystalline phase offers strength and rigidity.

### 4.1. Mechanical Properties of Recycled Plastics

Extensive research has been conducted to investigate the impact of recycling through injection molding, on the physical, chemical, and structural properties of plastics. However, when it comes to recycling by 3D printing, there is a notable scarcity of comprehensive literature in this domain. As a result, this section seeks to bridge the gap by drawing upon the insights and conclusions derived from the injection molding research. By extrapolating and adapting these findings, it is possible to gain valuable understanding and guidance for the effective recycling of plastics by 3D printing.

Section 2.4 highlights the impact of environmental factors and processing conditions on the mechanical properties of thermoplastics. Recycling involves heat and shear stresses that lead to degradation, while oxygen and moisture act as catalysts during processing, affecting the material’s properties. In injection molding, polymer degradation occurs through three primary process mechanisms: high temperatures required for melting, shear stresses during flow, and the residence time within the heated barrel. These parameters significantly influence the degradation kinetics of molten polymers. While reprocessing at high temperatures leads to significant thermal degradation, even lower reprocessing temperatures can induce degradation in polymers susceptible to mechanical degradation [220]. The processing conditions can alter the molecular structure of polymers through chain scission, crosslinking, and branching of polymer chains. High temperatures and shear stresses cause chain scission, reducing polymer chain length [221,222]. Additionally, crosslinking of polymer chains can occur in the presence of free radicals formed at elevated temperatures [30], which tends to decrease the mobility of polymer chains, making the material more brittle. The crosslinking in elastomers can be different from the crosslinking in thermosets or thermoplastics. While the crosslinks in elastomers are typically fewer and more flexible, the crosslinks in thermosets/thermoplastics are often numerous and highly rigid. With each successive recycling cycle, these changes become more pronounced as degradation accumulates. A higher number of reprocessing cycles results in increased brittleness, while an acceleration in chain scission contributes to earlier fracture occurrences after each cycle. Numerous studies have investigated the effects of mechanical recycling on the physicochemical and microstructural characteristics of thermoplastics [31,223,224,225,226,227]. Many of these studies have examined the impact of multiple recycling cycles on rheological and mechanical properties, consistently finding a decline in tensile strength, impact resistance, and elongation at break, indicating reduced strength, energy absorption capability, and stretchability of the polymer.

Additionally, an increasing number of recycling process cycles have been found to decrease the average molecular weight of the polymer chains, affecting their behavior, and influencing the melt flow properties of the recycled material [228,229,230,231,232]. Moreover, particularly in scenarios involving contaminants and microparticles, only a limited number of studies have delved into the implications of recycling on thermoplastics [233]. The presence of contaminants and microparticles has been reported to contribute to a reduction in creep resistance and fatigue properties of the recycled plastics [234].

### 4.2. Mechanical Properties of Recycled Plastics with Additive Manufacturing

Before delving into the evolution of recycled polymers through 3D printing, it is crucial to recognize that, in general, the mechanical properties of 3D printed parts are inferior to those produced through injection molding. Notably, the tensile strength, elastic modulus, elongation at break, and impact strength of 3D printed samples are significantly lower than those of injection molded samples [235].

In injection molding, a seamless and continuous structure is achieved, ensuring uniformity in both material composition and structural integrity. This process results in plastic parts with consistent properties throughout. On the other hand, 3D printing operates by building objects layer by layer, with the quality of adhesion between these layers governing the characteristics of the end product. The layering nature of 3D printing can introduce porosities and imperfections, leading to variations in structural properties. Consequently, mechanical testing often highlights the inferior properties of these dominant structural aspects rather than the material’s properties. Therefore, factors such as print speed, print orientation, sample size, layer dimensions and nozzle size might not have a direct impact on the fundamental material properties of the plastic being used in 3D printing [236,237,238]. However, they can still manifest differences in the overall properties of the printed parts during subsequent mechanical testing. The interplay of these factors can affect the bonding between layers, leading to variations in strength, durability, and other mechanical attributes. Therefore, establishing standards for the mechanical testing of 3D printed materials becomes significant for research literature comparison.

However, a significant factor governing the decline in mechanical properties due to recycling, as compared to using pristine materials, is degradation. In the field of 3D printing, based on our research, we have not come across any investigations that specifically analyze degradation mechanisms. Nevertheless, we can hypothesize that degradation mechanisms akin to those identified in injection molding components might also be operational in recycled 3D printed components. Consequently, our focus will be on investigating these mechanisms, aiming to enhance our comprehension of the potential degradation processes that could occur in recycled 3D printed components [239]. These processes involve a combination of thermomechanical degradation and thermal oxidation, with varying degrees of impact for each process. Moreover, even within the domain of 3D printing, two distinct processes exhibit differing levels of degradation: fused granular fabrication (FGF) and fused filament fabrication (FFF).

**Figure 10:**
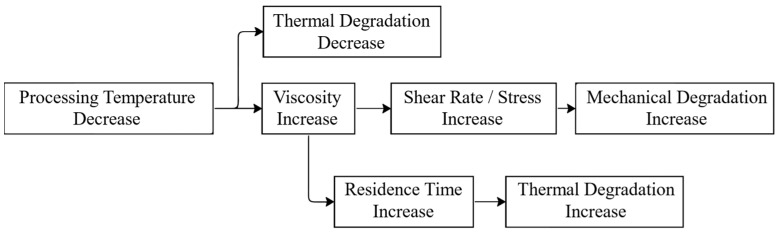
Effects of Decreasing Temperature on Viscosity and Residence Time: Relationship and Interactions.

In FGF (fused granular fabrication) 3D printing, the process involves three key mechanisms. First, thermo-oxidative degradation occurs due to the polymer undergoing melting at elevated temperatures. As a result, the material can experience chain scission. Secondly, during printing, the polymer is subjected to high shear forces in the screw extruder and nozzle, leading to additional mechanical degradation. The third mechanism involves residence time. Unlike injection molding, FGF 3D printing has a shorter residence time in the molten state. This is because the heating and melting process is localized to the immediate area where material is being deposited. Quantifying the exact contribution of each factor remains challenging. This complexity arises primarily from the interdependence of these variables, making it difficult to isolate their individual effects. Figure 10 illustrates a modification in one aspect, revealing the complex interconnection among these variables.

In comparison, FFF (fused filament fabrication) has similar degradation mechanisms to FGF, including thermo-oxidative degradation, shear forces during printing, and residence time considerations. However, FFF involves an additional step prior to printing—filament extrusion. This preliminary step demands dedicated heating and extrusion processes, which introduces further thermal and mechanical stresses to the material.

The study of the mechanical behavior of recycled polymers, specifically for 3D printing applications, is relatively limited. Anderson conducted a comparative study on 3D printed parts using virgin polylactic acid (PLA) and recycled PLA [240]. The mechanical testing results indicated that using recycled PLA for 3D printing is feasible, although certain mechanical properties exhibited variations. For instance, there was a reduction in tensile strength, an increase in shear strength, and a decrease in hardness. However, the tensile modulus of elasticity remained statistically unchanged. Notably, the recycled filament showed higher variability in the obtained results. In another study, the mechanical properties of 3D printed specimens using recycled PLA were investigated. The focus was on understanding the effect of interlaminar properties and short-beam strength. The findings revealed that the short-beam strength of specimens recycled once and twice was similar to that of virgin specimens. However, a third recycling process had a negative impact on the short-beam strength, leading to decreased strength and increased variability in the results [241]. Vidakis et al. explored various recycling approaches for acrylonitrile-butadiene-styrene [191], polyamide [165], polypropylene [189], high-density polyethylene [190], and polyethylene terephthalate glycol [242]. These investigations examined the impact of thermomechanical processing on the mechanical, thermal, and structural properties through multiple recycling cycles. The findings indicated that the materials exhibited increased stiffness and strength after the third and fourth round of recycling, but this effect was not observed after the fifth and sixth cycle. Furthermore, thermal analysis revealed no significant degradation until the fifth round of recycling. The results also showed that the crystallinity of high-density polyethylene and polyamide polymers decreased with an increasing number of extrusion cycles, while multiple extrusions predominantly resulted in crosslinking and branching, leading to an increase in mechanical properties.

### 4.3. Recycled Plastics Using Compatibilizers and Stabilizers

Additives play a pivotal role in plastic formulations, as they help maintain and modify the properties of polymers, enhance overall performance, and ensure long-term usability. Commonly employed additives effectively fulfill basic commercial requirements, primarily aimed at preserving the properties of plastics. In some cases, the implementation of specialized additives such as antioxidants, heat stabilizers, and light stabilizers becomes necessary to enhance durability, particularly in scenarios involving outdoor usage [243]. Moreover, in the context of recycling plastic waste, effective management of pre-damaged plastic is essential. This involves gaining insights into the material’s past, including the extent of degradation that has taken place, as well as identifying and quantifying any prior additives present. By strategically selecting and utilizing appropriate additives, the properties of recycled polymers can be optimized, promoting their effective reuse and contributing to a more sustainable approach to plastic waste management [244]. Table 6 outlines the primary advantages and disadvantages associated with each of the additives.

*Stabilizers* are intentional additives incorporated into plastics to protect the polymer against environmental factors such as heat, UV light, and mechanical stress. This ensures smooth processing and extends the service life of the recycled material in its intended application. Failure to evaluate the stability of used plastics before recycling can lead to a decline in their properties, resulting in the production of poor-quality products. In some cases, the substandard properties are mistakenly attributed to the recycled material alone, when the lack of proper stabilization is the actual root cause [244].

Stabilizers play a vital role in enhancing the properties and longevity of plastics, but their utilization in the recycling industry is not as widespread. Cost considerations often dissuade the recycling industry from incorporating stabilizers into their recycling processes, assuming that the virgin material already contains a sufficient amount of stabilizers. However, it should be acknowledged that the concentration of stabilizers in virgin plastics may decrease over time, especially during reprocessing [245]. This decrease can compromise their effectiveness, making it essential to consider adding stabilizers for recycled polymers. Unfortunately, a lack of information regarding the quantity of stabilizers in the plastic from its previous use poses a challenge for the recycling industry, particularly when repurposing the material for different applications. For instance, using plastic intended for indoor use in outdoor settings without weather-resistant stabilizers may lead to performance and appearance issues over time. Addressing these challenges and recognizing the importance of stabilizers in recycled plastics can greatly improve the overall quality and usability of recycled materials.

A recent review examined the importance of stabilizers in relation to the level of aging and oxidation in polymers, known as predamage [245]. Polymers that have undergone substantial damage during their previous use may necessitate higher concentrations of stabilizers compared to pristine materials. It becomes crucial to ensure the effective stabilization of polymers during their initial application to minimize degradation and ensure high-quality recycling outcomes. Although re-stabilization may incur additional costs, the benefits are manifold, including consistent processing, reduced downtime, improved material properties, and the ability to provide high-quality recycled grades suitable for a wide range of applications. Thus, the use of stabilizers is essential, even if the plastic’s next intended purpose does not demand top-tier quality. This practice maintains product quality for future recycling processes, contributing to a circular economy with high-quality materials. Moreover, the optimal stabilizer package for recycled plastics, in terms of both cost and performance, differs from that used for corresponding virgin plastics. This difference arises due to structural variations in the plastic, the presence of stabilizer residues from the initial application, and loose policies and standards [243].

In another recent investigation [246], the incorporation of a stabilizing additive into recycled polypropylene greatly enhanced its performance. The study utilized various characterization techniques such as spectroscopy, rheology, optics, and mechanical testing to assess the effects of recycling and additive incorporation. After subjecting polypropylene to 20 recycling cycles of mechanical processing, the study identified chain scission and oxidation of polymer chains as the main degradation processes. However, the addition of a small amount of a stabilizing additive proved to be highly beneficial. The properties of the recycled polypropylene significantly improved throughout the 20 reprocessing cycles. The additive acted as a hardener and facilitated crosslinking of the recycled polymer chains, resulting in enhanced performance.

*Compatibilizers*: Compatibilization involves the addition of a component which promotes physical or chemical bonds between the phases of a polymer blend. Compatibilizers are particularly important in polymer blends to enhance compatibility between different polymers. When two or more polymers with differences in chemical composition, polarity, and molecular weight are combined to achieve specific properties or characteristics, their blending can be hindered, leading to phase separation or weak interfacial bonding. Compatibilizers address this issue by having a unique chemical structure that allows them to interact and bond with both polymers. This interaction promotes molecular-level mixing and improves the adhesion between the polymer phases. When recycling polymer blends, it is often necessary to restore the original structure and stability of the material. This can be achieved through appropriate mixing processes and re-compatibilization of the blend. Similar to the initial preparation of the blend, it is crucial to create blends with consistent and stable properties during recycling and operation. Re-compatibilization plays a key role in achieving this objective as it helps to restore the original blend morphology and promote strong interfacial bonding between the different constituents. By employing re-compatibilization techniques, the recycled blend can maintain its desired characteristics and ensure reliable performance [107]. Furthermore, the use of compatibilizers presents potential opportunities for recycling mixed plastic waste, where sorting may be impractical. Compatibilization techniques can enhance the properties of mixed plastic waste, making mechanical recycling feasible [32].

The widespread adoption of polymer blend compatibilization is currently limited by cost-effectiveness concerns when compared to conventional waste management methods. Conventional approaches of diblock copolymers or in situ generated graft copolymers have a strong historical track record of effectiveness, but they may not be economically competitive with other waste management alternatives. Block copolymer structures are outside the scope of the present paper. For a thorough understanding, interested readers can refer to [247]. Nonetheless, there is considerable promise in using multiblock copolymer compatibilizers. Recent advancements in this area suggest that they could offer a viable solution to enhance the cost competitiveness of polymer blend compatibilization. Promising advancements have demonstrated that specially designed multiblock copolymers can significantly improve the blend compatibility of polymers, even when present in extremely small amounts, such as around 0.2 weight percent (wt%). This discovery presents an opportunity for a cost-effective option that may be feasibly integrated into industrial processes.

In the context of 3D printing, Zander et al. [195] processed blends of waste PET, PP, and polystyrene (PS) into filaments for 3D printing. They investigated the effects of compatibilization with SEBS elastomers on the properties of the filaments. Although the addition of SEBS elastomers did not significantly enhance the tensile strength, morphological analysis revealed improved bonding between the different phases of the blend. SEBS elastomers are thermoplastic materials with rubber-like behavior and full recyclability. Furthermore, the compatibilization process resulted in an increase in the glass transition temperature of the materials. This expanded the performance window of the filaments, making them more suitable for various applications.

Furthermore, despite the ongoing challenges, the use of compatibilizers presents potential opportunities for recycling mixed plastic waste. Where sorting is technically or economically impractical, the mechanical recycling of incompatible mixed plastic waste may become feasible through the use of compatibilization additives.

*Chain Extenders* are valuable additives, primarily used in polycondensation polymers such as polyesters or polyamides, to enhance the molecular weight of degraded polymers by reacting with their functional end-groups. This process, known as chain extension, involves linking low molecular weight materials with two or more functional groups to the carboxyl and/or hydroxyl end-groups of the polymer. The aim is to mend the polymer chains that may have broken during melt processing due to chain scissions [195].

In an ideal scenario with two end-groups per chain, difunctional additives can linearly increase the molecular weight. However, higher functionalities may lead to branching and cross-linking, depending on the concentration and functionality of the additive [245]. It is important to consider that some chain extenders might exhibit limited thermal stability, posing risks during multiple recycling cycles [248]. Furthermore, chain extension in certain cases may cause an excess of acid groups, accelerating degradation rates [30]. Careful selection of chain extenders is essential to maintain the desired properties of recycled polymers while mitigating potential issues during the recycling process.

## 5. Conclusions

The preceding sections cover the principles and steps of mechanical recycling of plastics and the recent idea of a more circular economy of plastics through distributed recycling by additive manufacturing (DRAM), and the mechanical properties of recycled polymers. Identified problems for plastic recycling generally stem from cost and quality as well as the highly technical nature of polymers and their applications. Plastic recycling often struggles to compete economically with the production of goods from virgin polymer sources. The problems of collection, sorting, and cleaning have all become more complicated, not less complicated, as more specialized plastics and applications were introduced in recent years. Problems also stem from polymer degradation due to environmental factors. As a solution to the recycling challenge, DRAM has emerged as a new approach with the benefits of decentralization, individual agency, and community participation. There remain questions to explore in establishing the feasibility of DRAM for various materials, scales, and contexts. The use of additives for stabilization and compatibilization are reviewed in this work and appear to be possible routes to enhance DRAM. Further study is needed on microstructural and mechanical property changes to plastics in DRAM systems and to optimize and control the fused filament and fused granule fabrication systems for maximum DRAM performance.

The centralized recycling facility has long been the adopted solution, but the current demands and complexities of logistics call for alternative approaches. The proposed DRAM approach, leveraging 3D printing and localized facilities, offers a promising solution by involving communities and addressing transportation challenges. Despite its potential, it is crucial to acknowledge that challenges will persist in the distributed approach. These challenges encompass various aspects such as technology access and knowledge, the establishment of community-based infrastructure and logistics, ensuring quality control of 3D printed components, managing material contamination and degradation, as well as the allocation of financial resources. To overcome these challenges, a multifaceted strategy is required, involving collaboration between governments, industries, research institutions, and local communities. Enhancing technological literacy and providing adequate training will empower communities to effectively utilize 3D printing technology. Investment in building and maintaining community-based recycling facilities and optimizing waste collection logistics will be vital for a successful distributed recycling system. Implementing stringent quality control measures and utilizing advanced sorting and cleaning technologies will ensure the production of high-quality 3D printed products from recycled materials. Additionally, research and innovation in materials science can lead to the development of more robust and sustainable recycled materials for 3D printing.

Furthermore, the benefits of this approach extend beyond natural resource conservation. Embracing 3D printing for localized recycling can support local economies, create job opportunities, and promote technological development in underserved regions. As technology becomes more accessible and user-friendly, the difficulties faced by inexperienced communities will diminish, paving the way for increased adoption globally. The DRAM approach presents a transformative path towards a greener, more efficient, and community-driven recycling system. With continued research and collaboration, society can unlock the full potential of 3D printing in plastic waste management, contributing to a more sustainable future for generations to come.

## Figures and Tables

**Table 1:** Comprehensive Summary of Published Review Articles.

Study Title	Aim/Objective	Comments/Key Findings	Reference
Mechanical recycling of packaging plastics: A review.	This review summarizes current methods and challenges in mechanically recycling five main packaging plastics. It also discusses ways to improve polymer blending in mixed plastic waste streams and uses for lower quality recyclate.	Across the five common types of plastic, changes in polymer chain length and mechanical properties remain a persistent challenge despite differences in the degradation mechanisms.	[30]
Recycling of waste from polymer materials: An overview of the recent works.	This study involves comparing the mechanical and chemical recycling techniques for various types of plastics, as well as analyzing the properties of polymers that have been mechanically recycled.	Mechanical recycling is the preferred and commonly used method of recycling compared to chemical recycling, which involves complex chemical treatments of the waste.	[31]
Mechanical recycling: Compatibilization of mixed thermoplastic wastes.	Approaches employed to achieve compatibility in blends of various thermoplastic waste.	Mechanical recycling of mixed plastic wastes can be viable if their properties are enhanced through compatibilization, but the stability behavior of the resulting materials must be considered before they can be utilized in the production of new goods.	[32]
Mechanical recycling of polylactide, upgrading trends and combination of valorization techniques.	This report provides an overview of the current state of mechanical recycling for PLA, with particular focus on a multi-scale comparison of various studies.	Out of all the recovery methods, mechanical recycling is the most cost-effective approach for PLA, but the recycled materials are typically used for lower-value applications due to inherent thermo-mechanical degradation.	[33]
Quality concepts for the improved use of recycled polymeric materials: A review.	This review explores new methods of mechanically recycling plastics to produce quality materials from waste streams.	Introducing a quality standard is crucial in plastic recycling. The biggest obstacle is finding a way to merge scientific understanding of the degradation and quality properties of recyclates with the design of an efficient upgrading process for each waste stream.	[34]
Mechanical and chemical recycling of solid plastic waste.	The current methods of polymer recycling, encompassing both mechanical and chemical recycling, are thoroughly described in this review.	Mechanical and chemical recycling are promising industrial techniques that can complement each other in closing the polymer loop.	[35]
Polymer recycling in additive manufacturing: An opportunity for the circular economy.	This short review focuses on the circular economy of materials and the recycling methods utilized in the polymer additive manufacturing process.	The development of recycled composites thorough fused deposition modeling (FDM) can lead to increased strength compared to that of the printed recycled polymer.	[36]
3D printing filament as a second life of waste plastics a review.	The main objective of this paper is to examine the existing literature concerning the use of recycled polymers in filament production for 3D printing, as an alternative to the current method of central selective plastic collection.	Traditional recycling methods have involved the use of large, centralized plants that produce low-value commodities, which results in high transportation costs. However, 3D printing presents new opportunities for recycling.	[37]
Plastic recycling in additive manufacturing: A systematic literature review and opportunities for the circular economy.	The focus of this study is to explore key themes within the six stages (recovery, preparation, compounding, feedstock, printing, and quality) of the distributed recycling by additive manufacturing chain proposed.	Limited efforts have been made regarding the recovery and preparation stages, whereas significant advancements have been made in the other stages to assess the technical feasibility, environmental impact, and economic viability.	[21]
Plastics recycling: challenges and opportunities.	The challenges that may arise during various stages of the recycling process were discussed, along with potential opportunities for enhancing recycling efforts.	Expanding the scope of recycling to include post-consumer plastic packaging, as well as waste plastics from consumer goods and end-of-life vehicles, can enhance the recovery rates of plastic waste and reduce the amount that ends up in landfills.	[2]
Fused deposition modelling approach using 3D printing and recycled industrial materials for a sustainable environment: a review.	This paper examines the sustainability of extrusion-based 3D printing materials, with a particular emphasis on the potential use of reusable and biodegradable materials.	Desktop 3D printing has the potential to advance plastic recycling through 3D printing.	[38]

**Table 2:** Recent Studies Investigating the Stages and Challenges of Plastic Recycling.

Recycling Stages	Management and Logistics	Waste Management	[39,40,41,42,43,44,45,46,47,48]
		Collection	[49,50]
		Supply Chain Modelling	[51,52,53,54,55,56,57]
	Mechanical Sorting	Sink-Float	[58,59,60,61,62]
		Froth Flotation	[63,64,65,66,67,68,69,70,71]
		Spectroscopy	[72,73,74,75,76,77,78,79]
		Magnetic Density Separation	[80,81]
	Shredding	Design and Modelling	[82,83,84,85,86,87]

**Table 3:** Cost Breakdown of the Operation of a Distributed Recycling Center.

Stage	Resource	Quantity	Time (Hours)	Cost ($)
Collection	Labor	N/A	N/A	Volunteer
Sorting	Labor	N/A	N/A	Volunteer
Cleaning	Water	10 Gallons	0.5	$0.02
Drying	Oven	1.88 kWh	6	$0.45
Shredding	Shredder	0.75 kWh	8	$0.14
**Total**				**$0.61**

**Table 4:** Efforts of Recycling Thermoplastic.

Topic	Material/Composite	Reference
Injection Molding	PP/Composite PP/Composite SEBS/PP ASA Composite Composite Nylon 12 PP/Composite	[179] [180] [181] [174] [182] [183] [184] [185]
3D Printing	HDPE PET Nylon 6 Composites PP HDPE ABS PET/Rubber PLA + Glass Fiber LPDE PET, PP, PS PET PP/Composite PLA	[186] [187] [188] [164] [189] [190] [191] [192] [193] [194] [195] [196] [197] [29]
Thermoforming	PP PET/Glass Fiber Composite	[161] [198] [199]

**Table 5:** Examples of Printing Parameters for FGF process Using the Gigabot X 2 XLT Printer.

Parameter	Value	Unit
Nozzle Diameter	2.85	mm
Layer Height	1.5	mm
Skirt Outlines	5	count
Bottom Heat Zone (T0)	185	°C
Middle Heat Zone (T1)	180	°C
Top Heat Zone (T2)	165	°C
Bed Temperature	60	°C
Printing Speed	900	mm/min
Travel Speed	6000	mm/min
3D Printer	Gigabot X 2 XLT	

**Table 6:** Key Attributes of Additives in Polymer Research.

Additive	Benefit	Drawback
Stabilizer	Prevents Degradation	Infeasible
Compatibilizer	Enhances Blend Compatibility	Infeasible
Chain Extender	Increases Molecular Weight	Thermal Instability
